# Complex Patterns of Genomic Admixture within Southern Africa

**DOI:** 10.1371/journal.pgen.1003309

**Published:** 2013-03-14

**Authors:** Desiree C. Petersen, Ondrej Libiger, Elizabeth A. Tindall, Rae-Anne Hardie, Linda I. Hannick, Richard H. Glashoff, Mitali Mukerji, Indian Genome Variation Consortium, Pedro Fernandez, Wilfrid Haacke, Nicholas J. Schork, Vanessa M. Hayes

**Affiliations:** 1 J. Craig Venter Institute, San Diego, California, United States of America; 2 The Scripps Translational Science Institute, Scripps Health and The Scripps Research Institute, La Jolla, California, United States of America; 3 Faculty of Medicine, University of New South Wales, Sydney, New South Wales, Australia; 4 The Garvan Institute of Medical Research, Sydney, Australia; 5 Division of Medical Virology, Department of Pathology, Faculty of Health Sciences, University of Stellenbosch, Tygerberg, South Africa; 6 Insitute of Genomics and Integrative Biology (CSIR), Delhi, India; 7 Division of Urology, Department of Surgical Sciences, Faculty of Health Sciences, University of Stellenbosch, Tygerberg, South Africa; 8 Department of Language and Literature Studies, University of Namibia, Windhoek, Namibia; 9 Department of Medical Sciences, Faculty and School of Health Sciences, University of Limpopo, South Africa; Dartmouth College, United States of America

## Abstract

Within-population genetic diversity is greatest within Africa, while between-population genetic diversity is directly proportional to geographic distance. The most divergent contemporary human populations include the click-speaking forager peoples of southern Africa, broadly defined as Khoesan. Both intra- (Bantu expansion) and inter-continental migration (European-driven colonization) have resulted in complex patterns of admixture between ancient geographically isolated Khoesan and more recently diverged populations. Using gender-specific analysis and almost 1 million autosomal markers, we determine the significance of estimated ancestral contributions that have shaped five contemporary southern African populations in a cohort of 103 individuals. Limited by lack of available data for homogenous Khoesan representation, we identify the Ju/'hoan (n = 19) as a distinct early diverging human lineage with little to no significant non-Khoesan contribution. In contrast to the Ju/'hoan, we identify ancient signatures of Khoesan and Bantu unions resulting in significant Khoesan- and Bantu-derived contributions to the Southern Bantu amaXhosa (n = 15) and Khoesan !Xun (n = 14), respectively. Our data further suggests that contemporary !Xun represent distinct Khoesan prehistories. Khoesan assimilation with European settlement at the most southern tip of Africa resulted in significant ancestral Khoesan contributions to the Coloured (n = 25) and Baster (n = 30) populations. The latter populations were further impacted by 170 years of East Indian slave trade and intra-continental migrations resulting in a complex pattern of genetic variation (admixture). The populations of southern Africa provide a unique opportunity to investigate the genomic variability from some of the oldest human lineages to the implications of complex admixture patterns including ancient and recently diverged human lineages.

## Author Summary

The Khoesan have received recent attention, as they are the most genetically diverse contemporary human populations. However, Khoesan populations are poorly defined, while archeological evidence suggests a once broader dispersal of click-speaking southern African foragers. Migrations into the regions populated by contemporary Khoesan involved agro-pastoral Bantu around 1,500 years ago, followed over a millennium later by the arrival of European colonists establishing a halfway station for a maritime route between Europe and the East, which led to unions between diverse global populations. Using almost a million genetic markers for 103 individuals, we confirmed a significant Khoesan contribution to five southern African populations. The Ju/'hoan show genetic isolation (early divergence from all other modern humans), carry no significant non-Khoesan contributions, and unlike most global populations lack signatures of gene-based adaption to agriculture. The !Xun show two distinct Khoesan prehistories; while comparable to the female-derived Khoesan contribution to the amaXhosa Bantu, the male-derived Bantu contribution to the !Xun most likely represents cultural-driven gender-biased gene-flow. Emanating largely from male-derived European ancestral contributions, the Basters showed the highest maternal Khoesan contribution, while the Coloured showed the largest within population and regional-associated variability. The unique admixture fractions of the two latter populations reflect both early diverged and recently diverged human lineages.

## Introduction

Southern Africa is home to populations carrying significant human genomic variation. The analysis of patterns of DNA variation, have placed modern human origins within Africa [1], with the most divergent contemporary lineages found in the indigenous Khoesan inhabitants of southern Africa [2]–[6]. Defined by their use of clicking languages and a foraging-based subsistence, contemporary Khoesan are largely restricted to the greater Kalahari regions of Namibia and Botswana. Representing a collection of isolated subpopulations with dwindling numbers and subpopulation extinctions, the Khoesan population identifier once represented a broader geographical dispersal reaching the most southern tip of Africa. Historical migrations into southern Africa including agro-pastoral Southern Bantu from a western/central African homeland beginning roughly 1,500 years ago [7], [8], followed over a millennium later by the arrival of European settlers and East-Indian slaves [9], shaped the ancestral contributions of contemporary southern Africans. These intra- and inter-continental contributions led to historical events that perpetuated population dispersals, isolations and assimilation between populations, ultimately giving rise to complex genomic admixture. The pattern of genomic variation in contemporary southern African populations thus resulted from unions between the most diverse genomes found within Africa to the least differentiated as represented by populations impacted by a severe founder effect (bottleneck) associated with the out-of-Africa dispersal [2], [10]–[13].

**Figure 1:**
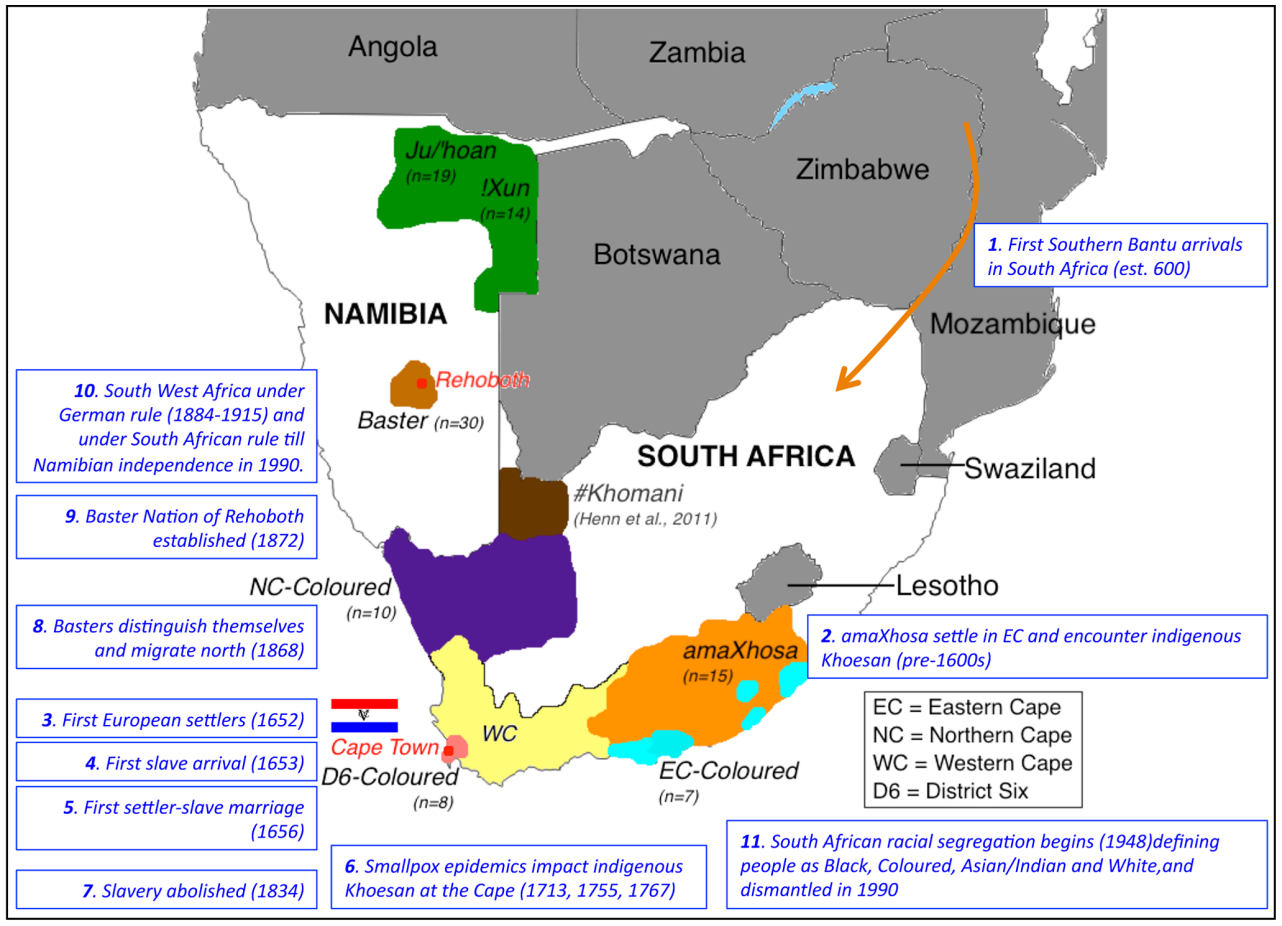
Map of southern Africa showing distribution of sampling per population identifier and significant historical events that likely shaped ancestral contributions. Study participants were sourced from two regions in Namibia and defined as Ju/'hoan (n = 19), !Xun (n = 14) (green) or Baster (n = 30, baige), while participants sourced within the boarders of South Africa included the amaXhosa (n = 15, orange) from the Eastern Cape and a geographical dispersal of the Coloured (n = 25). As the predominant population within the Western Cape (yellow), this region has contributed to the bulk of published Coloured data. The Coloured in this study reported a local heritage that included part of the Northern Cape (n = 10, purple), the Eastern Cape (n = 7, aqua), while the Western Cape Coloureds within this study were all originally from the hub of Coloured culture and population emergence, District Six (n = 8, peach). The geographical dispersal of the #Khomani is also indicated (brown). Relevant historical events that have shaped regional population admixture are indicated in chronological order.

Determining the ancestral origins of contemporary southern African admixture is limited by a number of factors including the availability of well-characterized subjects, limited availability of genomic data for appropriate founder populations, biases in current content genotyping arrays and analytical challenges. Lack of genomic data for southern African populations defined based on linguistics and culture broadly as Bantu and Khoesan, has perpetuated biases. To minimize these limitations, we leveraged genotype information from the largest current content array dataset that was available at the time the study was initiated in 2010, interrogating over 1 million genome-wide data points (Illumina HumanOmni1-Quad BeadChips). The 103 individuals in this study represent five southern African populations defined as Khoesan, specifically Ju/'hoan and !Xun, Southern Bantu, specifically amaXhosa, and European-initiated admixed populations, specifically Coloured and the newly described Baster population (Figure 1). At the time of submission there had been limited largely gender-specific analyses performed for pooled subsets of Southern Bantu [14], [15], while gender-specific [14], [16] and more extensive analysis for the Coloured had focused on non-regional sub-structure [17], [18]. While we previously considered the extent of whole exome diversity between two Ju/'hoan and a single !Xun, providing limited genome-wide analysis using the smaller 500 K Illumina arrays [5], no study had determined possible admixture contributions to these foraging-based populations. We merge our data with the only Khoesan-derived genome-wide dataset, the South African #Khomani [4]. The availability of globally relevant genomic data (published and from the Illumina iControl Database) provides a means to predict contributing migratory homogenous founder populations (specifically as a result of Bantu migration and European colonization), which most closely represent historical events that have impacted relations between southern African populations (Figure 1). In contrast, identifying indigenous founder contributions is more problematic. Contemporary Khoesan populations have either themselves experienced varying degrees of non-Khoesan contribution, or may not accurately represent the likely lost ancient ancestral lineages that once thrived along the southern coast of Africa at the time of non-Khoesan arrival. A major goal of our study was therefore to define a Khoesan population with negligible non-Khoesan contribution. Using anthropological, cultural, linguistic, as well as personal interactions within the remaining Khoesan communities of Namibia, the Ju/'hoan and !Xun were identified as likely candidates. Identifying early human divergence and unique forager-based genomic signatures, we further assess the significance of ancestral contributions within our study sample using multiple analytical approaches, while providing significant insights into the history of the region.

## Results

Before one can assess complex admixture fractions, it is critical to determine the validity of the study sample to be tested, paying particular attention to the potential for sampling biases, while assessing limitations within available genotyping content. Additionally, the identification of the most homogenous founder representative populations is critical. While the availability of globally relevant datasets allows for the assessment of non-regional founders, the identification of a homogenous indigenous founder population is currently not available. We provide evidence that supports the Ju/'hoan in this study as a likely representation of a homogenous Khoesan ancestral lineage. Using this data we provide multiple complimentary approaches to assess southern African admixture fractions.

### Limiting study biases

#### Sampling biases

All individuals self-identifying as Ju/'hoan or !Xun were extensively interviewed during six trips to the region over a period of three years (2008 to 2011) for further clarification see http://www.jcvi.org/cms/research/projects/southern-african-genome-diversity-study/. Individuals were excluded for reported relatedness and sourced from eleven remote sampling sites in Namibia (Figure S1). Culturally the groups display differing levels of alternative subsistence from hunting and gathering. While the Ju/'hoan maintain hunting rights, the !Xun demonstrate minimal subsistence crop cultivation. Both rely heavily on foraging and are impacted by western monetary influences in the form of government subsidies or limited earnings. Family units are generally small and geographical isolation is still apparent. While the Baster population was recruited within Namibia, the amaXhosa and Coloured were recruited within South Africa. Populations were selected based on historical significance that includes early Bantu or European arrival into the region, as well as accounts of Khoesan-derived contributions. All completed a questionnaire, which included self-identification via their maternal and paternal heritage, with place of birth used for regional subgroup classification, specifically Northern Cape (NC)-, Eastern Cape (EC)- and District Six Coloured (Figure 1).

#### Subject relatedness

Subjects were excluded if they reported at least one parent and/or one grandparent having belonged to an alternative population grouping, and included in the study based on heritage and geographical origins. Using 927,298 autosomal genotypes generated for 105 individuals, we performed identity-by-descent (IBD) allele sharing to assess cryptic non-self-identified relatedness among individuals. This resulted in the exclusion of two Ju/'hoan due to first cousin relations. No other subjects were excluded. Notably, three of the !Xun were originally from Angola and referred to themselves as Angolan !Xun or ‘Vasekela’. The genetic relationship of the self-identified distinction between Namibian and Angolan !Xun is currently unknown.

#### Subject source validation

Gender-specific markers are an ideal tool to rapidly ascertain likely ancestral contributions, providing a means of sample source validation using unique population-specific mitochondrial (mt)DNA and Y-chromosomal haplogroup identifiers. Khoesan-derived maternal and paternal lineages include the deepest rooting L0d and L0k mtDNA [14], [19], [20] and A and to a lesser extent the B Y–chromosomal haplogroups [21]. Mitochondrial haplogroup analysis (Table S1) showed 100% of the Ju/'hoan (n = 19) and 85.7% of the !Xun (n = 14) carry a L0d/L0k lineage, while 92.9% of the Baster (n = 30), 68% of the Coloured (n = 25) and 47% of the amaXhosa (n = 15) presented with a L0d lineage (notably lacking L0k representation). The non-Khoesan maternal contribution to the !Xun, Baster and amaXhosa were all associated with Bantu expansion, specifically L0a, L2 or L3′4 [7]. Regional diversity (Figure S2A) included highest L0d contributions in the NC- (90%), followed by D6- (62.5%) and lowest in the EC-Coloured (42.9%). In contrast Bantu-associated maternal lineages were significant contributors to the EC- (42.9%), with minimal contributions to the D6- (12.5%) and absent in the NC-Coloured. The M/N Eurasian mtDNA lineages were represented at low frequencies in all the Coloured subgroups, with highest contributions to the D6-Coloured.

Y-haplogroup analysis suggested a predominant Khoesan paternal contribution to the Ju/'hoan (11/14 males, 78.6%), specifically A2/A3b (10/11) and B2 (1/11), with remarkably minimal contribution to the !Xun (2/7 males A2-haplogroup, 28.6%). No data was available for the female contributing amaXhosa, while the Khoesan-derived paternal lineages were absent in the Coloured and Baster subjects. Non-Khoesan contribution to the !Xun was dominated by E1b1b (4/7 males, 57.1%). Defined by the M215 marker (previously E3b), E1b1b is reportedly a common contributor to East African Nilotic speakers [22]. A single Ju/'hoan presented with E1b1b, while a single Ju/'hoan and !Xun presented with the Bantu-derived west/central African predominant E1b1a8a and E1b1a7 lineages, respectively. The Baster and Coloured (Table S2) present with significant Eurasian paternal contribution defined by haplogroups R/I/G/N/O/J, at 92.3% (12/13 males) and 71.4% (15/21 males), respectively. Predominance of the largely Western European R1b haplogroup (12/27, 44.4%) was observed [7]. Regional distributions (Figure S2B) for the Eurasian-derived paternal haplogroups were highest for the NC- (83.3%), followed by the D6- (75%), and EC-Coloured (57.1%). Bantu-derived (non-Khoesan) paternal contributions were inversely distributed from the EC around the south to the northwesterly Basters. Unlike East African Nilotic E1b1b predominance in the Namibian Khoesan, E1b1a was the most common African-associated paternal lineage in the Baster/Coloured and linked to Bantu west/central African origins [7], [23]. Gender analysis confirms regional relevance of our study subjects.

#### Genotyping array content

Caution is needed in the interpretation of calculations of allele sharing between southern Africans using available genotyping arrays due to biases in content, which is typically derived from studies on recently diverged populations. Determining the overall mean percentage autosomal heterozygosity for the 927,298 variants provides an estimate of bias for the largest array content at the time. The assumption is that percentage heterozygosity should decrease as one moves away from human origins in Africa, factoring in additional contributions such as time since dispersal and admixture events. The most significant contributions to current array content including European, Asian and Yoruba, show heterozygosity percentages of 27.7% (n = 175), 25.7% (n = 44) and 29% (n = 90), respectively. The amaXhosa was well represented at 28.5% heterozygosity, although whole genome sequencing for a single Southern Bantu individual suggested overall heterozygosity reaching almost 60% [5]. The lower mean percentage observed for the Ju/'hoan (23.4%) and !Xun (25.2%) draws attention to array limitations for ancient lineages. The highest percentages observed for the Coloured and Baster (30% each) is as expected for populations with diverse ancestral contributions. Mindful of these limitations, we leveraged multiple approaches to assess Khoesan contributions and homogenous lineage representation.

### Defining homogenous founder representation

#### Principal components analysis (PCA)

**Figure 2:**
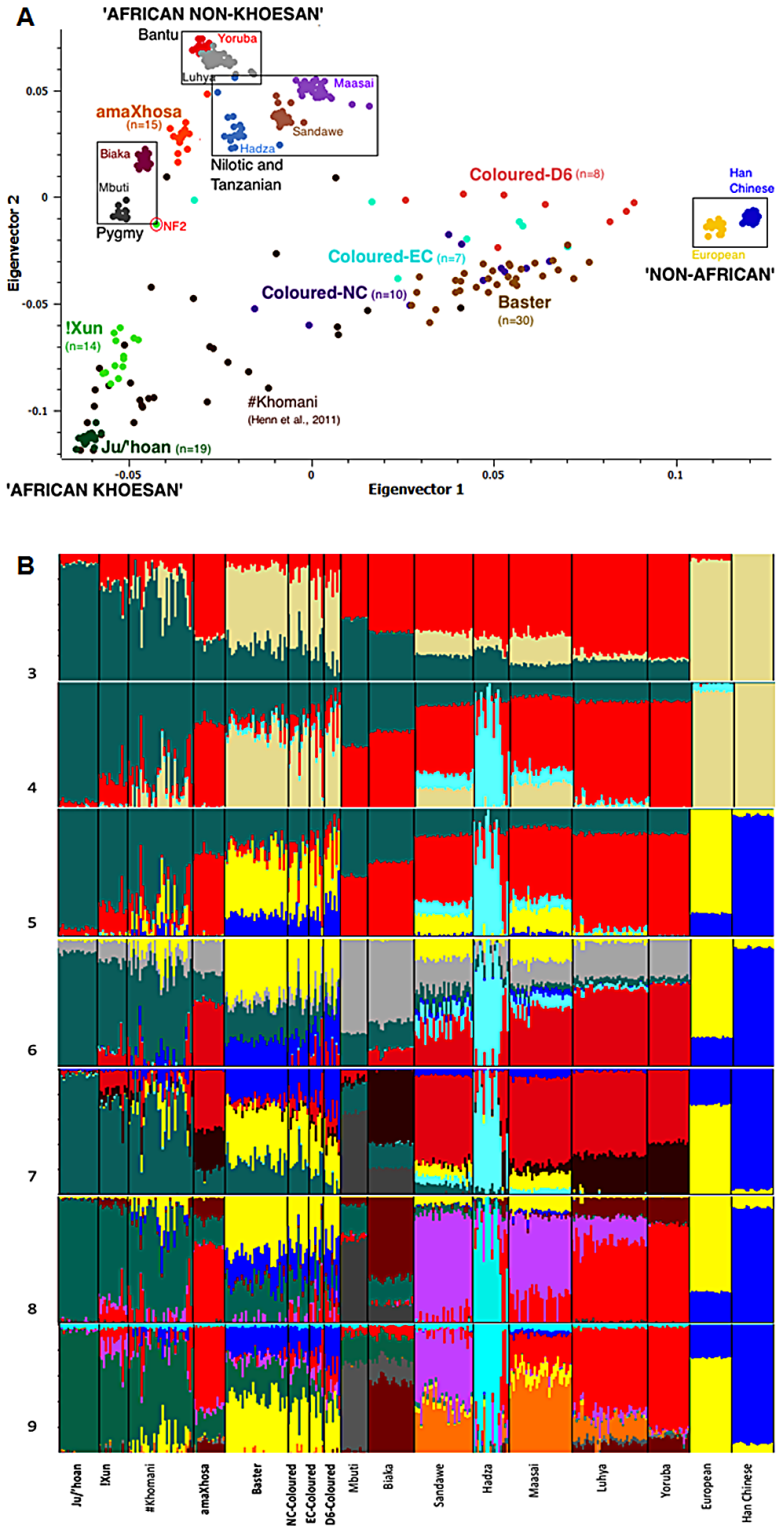
Population substructure within the five southern African populations (n = 103) in relation to 10 globally relevant populations (n = 237) for 33,207 overlapping autosomal markers. (A) Principal Components Analysis (PCA) clustering of the Yoruba, Ju/'hoan and Han Chinese/Europeans at the outer extremes of Coloured and Baster dispersal is suggestive of ‘African non-Khoesan’, ‘African Khoesan’ and ‘non-African’ substructure, while the amaXhosa and !Xun form tight clusters representing their Bantu and Khoesan ancestral roots, respectively. (B) STRUCTURE analysis (K = 3 to K = 9) further defines homogenous ancestral contributions defined in this study as ‘Khoesan’ (Ju/'hoan, green), ‘African proto-Bantu’ (Yoruba, red), Hadza (light blue), ‘Asian’ (Han Chinese, blue), European (yellow), and ‘Pygmy’ (light gray), the latter further defined (K = 7) as Mbuti (dark gray) and Biaka (maroon), Sandawe (light purple) and ‘Nilotic’ (Maasai, orange).

We used PCA to provide an interpretable assessment of homogeneity and geographic origins of our study subjects (n = 103) in relation to globally relevant and platform-matched publically available data (n = 237). Merged data allowed for the interrogation of 33,207 overlapping genetic variants within 15 population identifiers. PCA shows clustering of the amaXhosa close to their Bantu ancestral roots, the Ju/'hoan and !Xun (exception NF2) show clustering defined by their Khoesan ancestral heritage, while the Coloured and Baster, as well as the published #Khomani, are highly admixed, suggesting ‘African non-Khoesan’, ‘African Khoesan’ and ‘non-African’ ancestral fractions (Figure 2A). The Yoruba, Ju/'hoan and European/Han Chinese form tight clusters at the outer extremes of these ancestral contributions, respectively.

#### STRUCTURE analysis

Further insight into the relationships of the southern African populations was provided using STRUCTURE analysis [24] (Figure 2B). Assuming three population clusters the most significant ancestral contributions were represented within the Ju/'hoan (92.1% ‘African Khoesan’), Han Chinese/European (99.8%/95.2% ‘non-African’) and the Yoruba (81.9% ‘African non-Khoesan’), assuming four populations the Hadza (66.2%), a Tanzanian hunter-gatherer population, form an independent ancestral contribution, while for five contributing populations the Europeans (81.8%) and Han Chinese (94.9% ‘Asian’) become differentiated. Based on assessment of the log likelihood for probability of the data generated, assuming five population clusters provides the ‘best fit’ model. Extended differentiation into six ancestral clusters suggests a central African ‘Pygmy’ derived fraction rather than a ‘Khoesan’ contribution throughout Africa, while seven ancestral clusters further differentiates the ‘Mbuti’ and ‘Biaka’ derived Pygmy contribution. Observing a significant ‘Khoesan’ contribution maintained within the Pygmy and to a lesser extent the Sandawe, the ‘Biaka’ ancestral cluster shows significant contribution to the Bantu nations, including the amaXhosa (Southern Bantu), Luhya (Eastern Bantu) and Yoruba (proto-Bantu). Lack of a ‘Biaka’ signature within the Ju/'hoan provides further validation for ancestral homogeneity, while lack of significant ‘Biaka’ ancestral signatures within the Maasai (Nilotic), and the Sandawe and Hadza click-speaking east African populations provides a distinction between Bantu and non-Bantu. The latter observation is maintained when assuming eight ancestral population clusters, with the addition of a ‘Sandawe’ predominant cluster. The Sandawe share ancestral contributions with the Maasai and to a lesser extent the Luhya, which when assuming nine ancestral clusters is defined as a ‘Nilotic’ (Maasai) ancestral contribution to the Sandawe and Luhya. The latter defines an ancestral distinction between the proto-Bantu (Yoruba) and the Eastern Bantu (Luhya) speakers. Interestingly the non-Khoesan African contribution to the !Xun appears to be of Bantu and Sandawe ancestral origins. STRUCTURE analysis was therefore unable to confirm a Nilotic contribution to the !Xun as suggested by Y-chromosomal analysis.

#### Generalized analysis of molecular variance (GAMOVA)

GAMOVA is a powerful tool to quantify the explanatory power of population identifiers based on genome variability among a set of individuals grouped into categories based on those identifiers [25]. We used this approach to test the hypothesis that the population labels assigned to each individual was consistent with genotypic differences between those individuals. Performing GAMOVA for an expanded sample set of 588 individuals using 24,402 overlapping autosomal markers cleaned and pruned for linkage disequilibrium (LD), identified the European, Han Chinese, Yoruba and Ju/'hoan as presenting with the least within-group heterogeneity (Table S3). These population identifiers most likely represent a homogenous ancestral contributions defined in this study as ‘European’, ‘Asian’, ‘African non-Khoesan’ and ‘African Khoesan’, respectively.

### Validation of Ju/'hoan as an ancient forager human lineage

Our data suggests that the Ju/'hoan represent the most likely homogenous contemporary Khoesan population. Two factors that set the Khoesan apart from other global populations include early divergence and forager substituted by hunting existence. We use genomic data to look for signatures that differentiate the Ju/'hoan in this study based on these criteria.

#### Phylogenetic analysis

**Figure 3:**
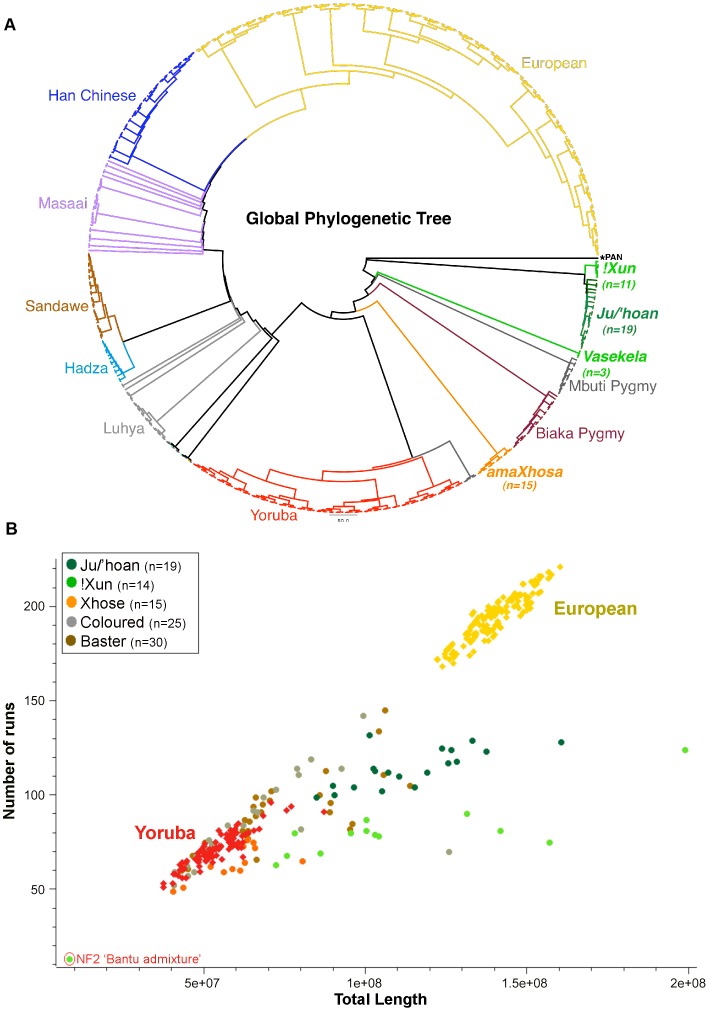
Relatedness and demographic history of the Ju/'hoan to global populations defines early divergence and genomic impact of forager existence. (A) Circular Neighbor Joining phylogenetic tree for 24,402 LD-pruned autosomal markers after merging our data with global population data for a total of 521 samples from 14 populations, with the Pan genome as the outgroup. We confirm early divergence of the Ju/'hoan and report independent branching of the Angolan !Xun. (B) Plot of total length of ROH against number of ROH (>500 kb) for each study sample against European and Yoruba using 716,734 markers (367 samples). Our foraging groups show smaller overall ROH lengths than the Europeans, yet longer than the Yoruba, suggesting small effective population sizes of a likely ancient population with minimal to no impact from a dramatic bottleneck.

We used the merged dataset for 521 individuals from 14 populations to generate Neighbor Joining (NJ) phylogenetic trees on the basis of identity by state (IBS) similarity of the individuals, with the *Pan* genome as the outgroup. Represented as a rooted circular tree (Figure 3A), we confirm genetic distinction of the Ju/'hoan and !Xun as the earliest known diverged human lineage, with the Ju/'hoan showing an earlier split. Interestingly, the three Angolan !Xun (Vasekela) form an independent branching from the Namibian !Xun.

#### Runs of Homozygosity (ROH)

Determining the fraction of ROH provides a demographic history of a population, which differs significantly between small forager populations and large agricultural communities. We plotted the total length of ROH against the number of ROH (greater than 500 kb) for each population identifier in our study against the Yoruba, representing an outbred African ancestral agricultural lineage, and Europeans, representing inbreeding impacted by genetic drift and a significant bottleneck [26] (Figure 3B). Like the Yoruba, the amaXhosa present with shorter and fewer ROH as expected for a large outbred agricultural-based community. In contrast, the forager societies in this study are traditionally made up of small family units, having moved with the seasons and available resources within confined, sparsely inhabited geographical regions. The smaller overall ROH lengths compared with the Europeans, yet longer ROH than those observed in the Yoruba, concurs (although does not validate due to multiple possible contributing factors that impact ROH) with a demographic history that is; (i) early diverged, (ii) arguably not impacted by a significant bottleneck associated with major migration event and (iii) historical maintenance of smaller forager populations. Complex admixture is the likely contributor to ROH shortening within the Coloured and Baster populations.

#### African forager PCA

The conversion to agriculture over 10 thousand years ago was a global event [27], resulting in a dramatic decline in forager or true hunter-gatherer societies. A handful of forager peoples still live within geographically defined regions within Africa. Besides the Ju/'hoan and !Xun from the greater Kalahari semi-desert region of Namibia, genomic data has been made available for the more southerly #Khomani (southern African Khoesan), the Mbuti and Biaka (Central African Pygmy) and the Sandawe and Hadza (East African Tanzanian Click-speakers) [4]. PCA analysis of merged data (n = 144) shows an independent clustering of the Ju/'hoan and !Xun from the Tanzanian and Pygmy groups (Figure S3). Like the single !Xun NF2 (recent Bantu admixture), the #Khomani although essentially southern African Khoesan show broad dispersal suggesting differing degrees of recent non-Khoesan contributions.

#### Target signatures of foraging

The event of agriculture undoubtedly impacted the genomic differences between contemporary global populations, contributing to significant selective pressures on the human genome [3]. Functionally relevant genomic signatures that distinguish ‘forager’ from ‘non-forager’ (or non-agriculturalist from agriculturalist) have been suggested and tested in this study (Figure S4). Significant adaptations to agriculture have been associated with pathogen emergence [28] and dietary challenges [29]. Unlike their Bantu neighbors, the Ju/'hoan demonstrate close to fixation for the pre-agricultural malaria associated Duffy allele (Figure S4A), while fixation for the ancestral non-truncating *PLRP2* variant appropriately reflects forager root-based diets and lack of adaption to cultivated grains (Figure S4B). While rapid identification and eradication of dietary toxins is essential for forager survival, this need is no longer a selective advantage within agricultural societies. Three nonsynonymous *TAS2R38* variants forming the non-taster (C-T-T or AVI) haplotype have been associated with an inability to sense a bitter taste from the compound phenylthiocarbamide [30]. Non-taster homozygosity, a selective disadvantage for forager societies, was absent in both our Ju/'hoan and !Xun (Figure S4C). In parallel, four nonsynonymous *NAT2* variants have been associated with acetylation of exogenous chemicals found in the diet and environment, with the fast acetylation haplotype (G-T-G-G) advantageous to forager societies [31]. All the Ju/'hoan presented with G-T-G-G either homozygously (68.4%) or heterozygously (31.6%) (Figure S4D). In contrast, agricultural societies face new toxic challenges exasperated by industrialization. While flavin-containing monooxygenases (FMOs) function to metabolize a variety of foreign toxins, FMO2 has been shown to increase the toxicity of thiourea found in many industrial and medical products, including adverse reactions to treatments used for tuberculosis. A truncating *FMO2* variant causing protein inactivation [32], has reached fixation in Eurasians [33]. We found the active full-length ancestral allele to be present at the highest reported global frequencies in the Ju/'hoan (78.9%) and !Xun (78.6%) (Figure S4E). Behavioral phenotypes essential for foraging existence include the need for an instantaneous response to adverse stimuli. The ancestral allele for a nonsynonymous variant in the gene *COMT* has been associated with increased enzymatic activity [34] and a need to react to adverse emotional stimuli [35], while the derived allele has been associated with the emergence of attention-related tasks [36], [37]. The ancestral allele was fixed in the Ju/'hoan (Figure S4F). The Ju/'hoan in this study demonstrate significant maintenance of known forager-based genomic signatures.

#### Unique signatures of foraging

Assuming homogenous forager and agriculturalist representation by the Ju/'hoan and Yoruba, respectively, provides an ideal opportunity to identify novel genomic events that distinguish independent evolutionary and adaptive pathways, while further assessing for early divergence through ancestral maintenance within the Ju/'hoan. Comparing allele frequencies for 70,733 LD-pruned variants, identified 2,687 AIMs with a −log10 p>5 (Figure S5). We found 32 to be fixed within the Ju/'hoan, yet under mutational pressure within the Yoruba. Comparisons with the *Pan* genome resulted in 30 corresponding allelic positions, of which 25 (83%) were ancestral and five (17%) derived in the Ju/'hoan (Table S4). Six of the 17 most significant loci-relevant (26 alleles −log10 p>14, Table S5) and 12 of the 33 amino acid changing AIMs (Table S6) were found within gene regions previously associated with chemical dependency to tobacco (as defined by the Genetic Association Database; http://geneticassociationdb.nih.gov/) [38]. SIFT analysis (http://sift.jcvi.org) defined three variants as potentially ‘damaging’, including tobacco-associated loci *PKD1L2* (V308G) and *EIF4G3* (Q500H). The 1,162 AIMs found to lie within a gene region using the MetaCore pathway analysis and data mining tool from GeneGO (http://www.genego.com/), were further ranked by significance based on gene ontology to biological processes (Table S7), molecular functions (Table S8), cellular localization (Table S9), diseases associations (Table S10) and pathway map folders (Figure S6). Diseases associated with genes representing significant differences between the Ju/'hoan and the Yoruba include autoimmune or immune diseases (specifically associated with the nervous system), psychiatric or mood disorders, followed by tobacco use disorder. While wound repair, blood clotting, inflammatory and immune response, arguably critical for forager survival, all occur within the top ranking significantly enriched pathway map folders, we once again found the nicotine action pathway to be implemented. Significantly enriched pathways that may reflect foraging-based characteristics of adaption to extreme heat include maintenance of thermal homeostasis through controlling sweat rates (cystic fibrosis disease) and heart rates (vasodilation/vasoconstriction, cardiac hypertrophy and myogenesis regulation) [39], and maintenance of fluid balance as a result of minimal availability of water (calcium signaling and diuresis).

### Determining Southern African admixture fractions

#### Estimations of similarity

Calculating Fst values using a supervised Admixture run and identity by state (IBS) distance sharing using PLINK (the latter for within and between population identifiers) was used to estimate population identity clusters for similarity (or dissimilarity) for 588 individuals from 15 populations using 24,402 markers (Table S11). The Ju/'hoan exhibit the greatest within group *similarity* (0.224), show the greatest *similarity* with the !Xun (IBS 0.240; Fst 0.047) and #Khomani (IBS 0.249; Fst 0.045) and the greatest *dissimilarity* with non-African populations, namely Han Chinese (IBS 0.317; *Fst* 0.188) and European (IBS 0.311; *Fst* 0.151). Although clearly both Khoesan, compared with the Ju/'hoan, the !Xun show greater *similarity* with Bantu populations (specifically amaXhosa, Luhya and Yoruba), while the #Khomani show greatest *similarity* with non-Africans (specifically European and Han Chinese). The amaXhosa exhibit the greatest *similarity* with the West and Eastern Bantu populations (IBS 0.274 each; Fst 0,032 Yoruba and 0.033 Luhya) and Khoesan populations (!Xun IBS 0.272, Fst 0.071; Ju/'hoan IBS 0.274, Fst 0.091). The Baster and Coloured show the strongest overall between-population *similarity* (IBS 0.281; Fst 0.02) and the largest *similarity* for all populations tested with Europeans (Baster IBS 0.283; Fst 0.034; Coloured IBS 0.286, Fst 0.037).

#### Ancestral contributions

STRUCTURE analysis assuming five ancestral clusters (the best fit model based on log likelihood estimations) and considering clusters defined as ‘African Khoesan’ (Ju/'hoan), ‘African non-Khoesan’ (Yoruba), ‘European’, ‘Asian’ (Han Chinese) and ‘Hadza’ (Figure 2B), provides an estimation of ancestral fractions (Table S12). No population in this study showed any ‘Hadza’ ancestral contribution. The !Xun and #Khomani appear to share 77.1% and 70.6% ‘Khoesan’ ancestral contribution with the Ju/'hoan, respectively. Unlike the Ju/'hoan, the !Xun show an ancient largely uniformly distributed ‘African non-Khoesan’ contribution (20.5%), while confirming recent Bantu admixture within a single individual NF2 (47%). The #Khomani show more complex recent admixture including ‘African non-Khoesan’ (13.9%), ‘European’ (11%) and minimal ‘Asian’ (3.7%) contributions. The amaXhosa share roughly 63.6% ‘African non-Khoesan’ ancestral contribution with the Yoruba with a significant uniform ‘Khoesan’ (35.4%) contribution suggesting an ancient admixture event. The Baster and Coloured show largely a non-African contribution, specifically ‘European’ (48.4% and 38.5%, respectively) and ‘Asian’ (17.1% and 19.9%, respectively), with significant ‘Khoesan’ contribution (28.5% and 25.3%, respectively). The ‘African non-Khoesan’ contribution appears to be more significant in the Coloured (15.5%) than the Baster (5.7%). Geographical-defined contributions to the Coloured include an increased ‘Asian’ (29.4%) and reduced ‘Khoesan’ (14.4%) contribution to the D6-, highest ‘Khoesan’ (33%) and lowest ‘African non-Khoesan’ (10.1%) to the NC-, and highest ‘African non-Khoesan’ (21.1%) and lowest ‘Asian’ (14.7%) contributions in the EC-Coloured (Figure S7).

#### Ancient admixture fractions

**Figure 4:**
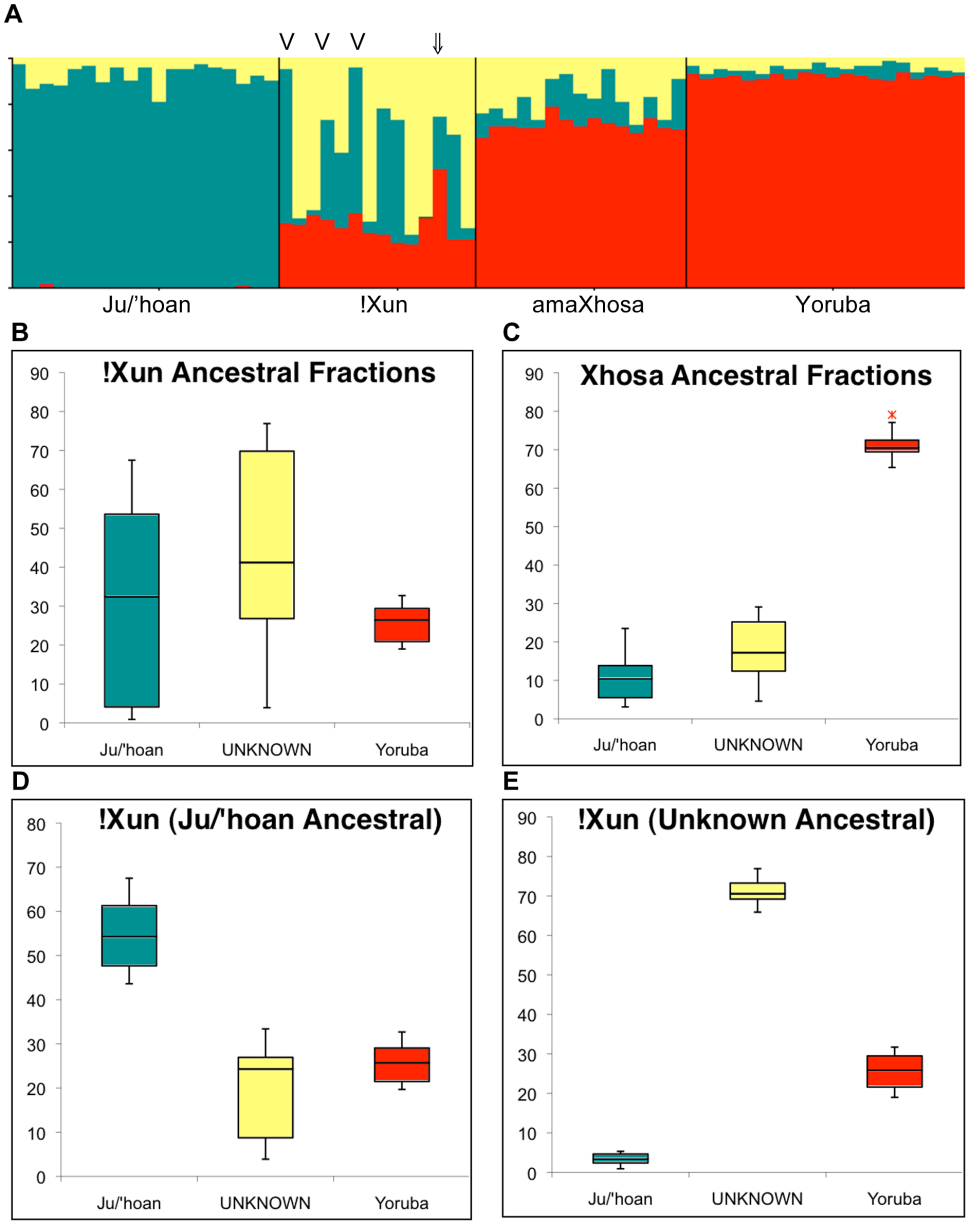
Ju/'hoan-Yoruba ancestry informative markers (AIMs) defined ancestral contributions to the !Xun and amaXhosa, providing evidence for two distinct !Xun lineages with differing ancestral contributions. (A) STRUCTURE analysis for 2,687 Ju/'hoan-Yoruba AIMs identifies a third ‘unknown’ population cluster when assuming three ancestral populations. (B) Ancestral contributions to the !Xun shows a diverse contribution of a Ju/'hoan and unknown likely Khoesan ancestral fraction and a constant Bantu-derived fraction. (C) Ancestral contributions to the amaXhosa demonstrate more even contributions. (D) Based on ancestral fractions the !Xun are further classified as Ju/'hoan ancestral (n = 6) and (E) unknown ancestral, suggesting two unique !Xun lineages, each with significant Bantu ancestral contributions. The single admixed !Xun (NF2 ⇓) and the three Angolan !Xun (V) are indicated.

The !Xun and amaXhosa show ancient African ancestral contributions defined as ‘non-Khoesan’ and ‘Khoesan’, respectively. Expanding the STRUCTURE analysis from five to nine ancestral population clusters (Figure 2B) confirms complex population substructure within Africa. The non-Khoesan contribution to the !Xun appears to be ancestrally related to the ‘proto-Bantu’ (12.7%) and ‘Sandawe’ (10.9%), while refuting any significant ‘Nilotic’ contribution. A ‘Sandawe’ related ancestral contribution was additionally observed within the Coloured (6.2%), Baster (4.7%), amaXhosa (4.4%) and #Khomani (2.8%), while being absent from the Ju/'hoan. Inability to further define Khoesan-derived substructure (assuming further ancestral clusters, data not shown) was likely contributed by array content biases. We therefore applied STRUCTURE analysis for the 2,687 previously described Ju/'hoan versus Yoruba AIMs to further evaluate the Khoesan ancestral contributions to the !Xun (∼72%) and amaXhosa (∼28.5%) (K = 2, Figure S8). Assuming three independent ancestral populations resulted in the replicable identification of varied ancestral contributions to the !Xun (Figure 4A). The identification of a significant ‘unknown’ fraction appears to be the largest contributor to the !Xun, average 43.6% compared to 28.9% Ju/'hoan contribution (Figure 4B), yet presents with extreme individual contributing variability (range 3.9%–76.9%) in comparison to the amaXhosa (Figure 4C). Notably, the non-Khoesan contribution to the !Xun is relatively maintained. We speculate that the Khoesan ancestral diversity likely reflects the broad usage of the !Xun identifier. For example we note the Angolan !Xun (Vasekela) carry a predominant Ju/'hoan (average 58.2%) over the ‘unknown’ (average 11.8%) ancestral fraction, suggesting a genomic-based distinction between the Angolan and Namibian !Xun. Furthermore, distinction of these ancestral fractions identified six !Xun as Ju/'hoan ancestral (Figure 4D) and six !Xun as ‘Unknown’ (or non-Ju/'hoan) ancestral (Figure 4E). We cannot ignore that the distinction of two distinct !Xun sub-populations on the basis of AIMs specific to Ju/'hoan may create biases, but we speculate that these biases are more likely to be in the direction of not finding evidence for additional substructure.

#### Significance of ancestral fractions

**Figure 5:**
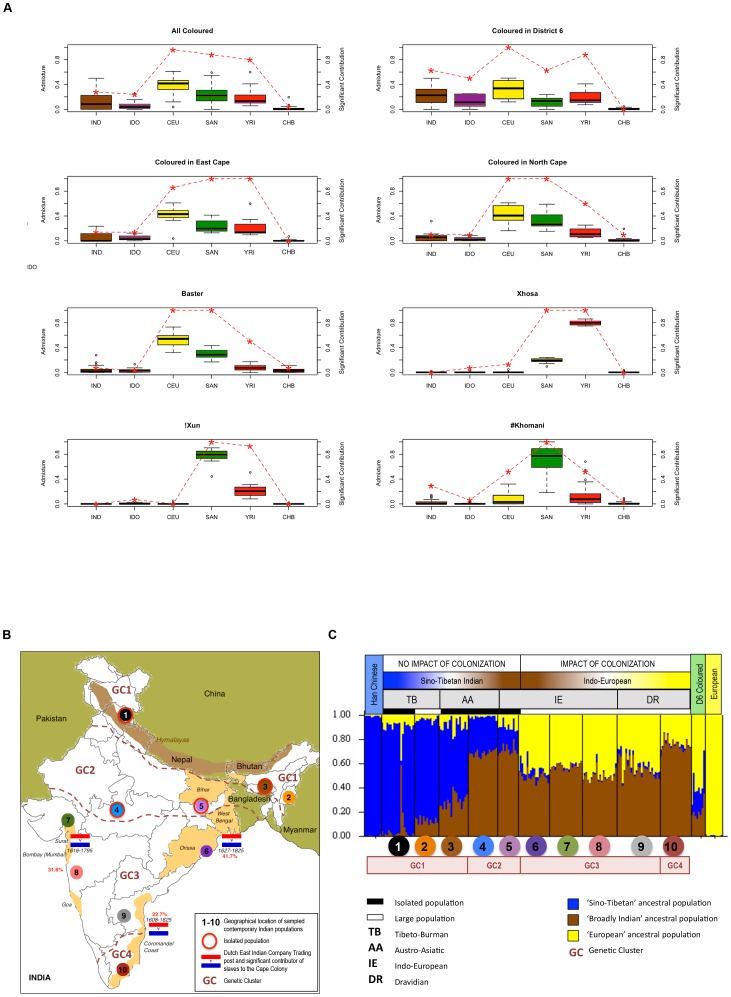
Estimation and significance of ancestral contributions to southern African populations, including further clarification of recent Asian contributions. (A) Boxplots show the estimated ancestral fractions based on ADMIXTURE analysis for six population clusters defined as IND (Indian), IDO (Indonesian), CEU (European), SAN (Ju/'hoan), YRI (Yoruba) and CHB (Han Chinese) for the !Xun, #Khomani, amaXhosa, Baster and regionally defined Coloured (3,725 markers). The red dashed line depicts significance of ancestral contributions. A value of 1.0 indicates that each individual has a significant contribution from the respective ancestral population, a value of <1 indicates that the ancestral contribution is observed only in a proportion of the individuals, while a value of 0 indicates complete absence. (B) Map of India representing the sampling sites for 10 population identifiers and four regionally relevant genetic clusters (GCs). Flags depict the geographical location and date of Dutch East Indian Company (VOC) trading posts, with percentage in red depicting the estimated slave contribution to the Cape Colony. (C) STRUCTURE analysis of 9,317 LD-pruned autosomal markers for three ancestral populations distinguishes between a ‘Sino-Tibetan’ and ‘Indo-European’ population cluster, the latter significantly impacted by colonization of India. This data suggests that both clusters contributed to slavery at the Cape.

To provide a more accurate assessment of the ‘Asian’ ancestral contribution, we expanded our analysis to include published data that reflects historical accounts of Indian (36.4%) and Indonesian (31.5%) derived slave contributions [40]. While we have previously alluded to the unlikely ancestral contribution of Han Chinese to the Coloured [17], we provide evidence for lack of the East Asian specific ‘dry earwax’ (*ABCC11* rs17822931-AA) [41] and the ‘alcohol-induced flush’ genotypes (*ALDH*2 rs671 A-allele) [42] in our study subjects. ADMIXTURE analysis [43] of our data merged with that published for 20 Indonesians [17] and 179 Indians [44]–[46] was used to establish significance based on ancestral representation in each individual within a population identifier assuming six population clusters with the separation of Han Chinese, Indonesian and Indian (Figure 5A and Table S13). The inclusion of these datasets however dramatically reduced the total number of available markers for interrogation to 3,725. We confirm significance of ancient ‘African non-Khoesan’ (Yoruba) and ‘Khoesan’ (Ju/'hoan) signatures to 93% of the !Xun and 100% of the amaXhosa, respectively. ‘Khoesan’ contribution was also significantly represented in all study participants classified as !Xun, #Khomani, Baster, NC-Coloured and EC-Coloured. Differences in ‘Khoesan’ contribution to the NC- and EC-Coloured compared with the D6-Coloured was significant (*p*-value 0.002 and 0.04, respectively). All the EC-Coloured showed a significant ‘African non-Khoesan’ contribution, while the ‘European’ contribution reached significance within the Coloured (96% overall), Baster (100%) and in about half the #Khomani. We confirm negligible Han Chinese contribution to regional populations, with significant ‘Indian’ contribution to 29% of the #Khomani, while overall contributing 28% ‘Indian’ and 24% ‘Indonesian’ significance to the Coloured. The ‘Indian’ and ‘Indonesian’ fractions were contributed overwhelming to 63% (*p*-value 0.04 compared to NC- and EC-Coloured) and 50% (*p*-value 0.03 compared to the NC-Coloured) of the D6-Coloured, respectively. Previously we demonstrate the Indonesian contribution to include Makassar, Bugi and a lesser extent Javanese [17]. We further establish the Indian contribution with a focus on the District Six Coloured.

#### Indian admixture fraction

Abolished in 1980, District Six was once the center of the Coloured nation at the heart of European settlement and Dutch-East Indian Company (VOC) slave trade [47]. Sourced from 10 populations, inclusion of the Indian dataset was selected based on geographical distribution and relation to historically relevant VOC settlements (Figure 5B). STRUCTURE analysis was performed on the merged dataset (9,317 markers) including the D6-Coloured, iControl Han Chinese and European representatives, and the Indian dataset. Assuming three ancestral populations (Figure 5C), we describe a ‘Sino-Tibetan’ (Han Chinese and Tibeto-Burman language grouping), ‘broadly Indian’ and ‘European’ ancestral contribution resulting in geographically significant genetic clusters (GCs). Defined as GC1 with a predominantly ‘Sino-Tibetan’ (∼76%) and lesser ‘broadly Indian’ (∼16%) contribution, GC2 with a predominantly ‘broadly Indian’ (∼70%) and lesser ‘Sino-Tibetan’ (∼25%) contribution, GC3 with a predominantly ‘broadly Indian’ (∼53%) and a significant ‘European’ (∼44%) contribution, and GC4 with a predominantly ‘broadly Indian’ (∼75%) and a less significant ‘European’ (∼23%) contribution than the GC3 cluster. The genetic relatedness between the Tibeto-Burman populations and Han Chinese, and the Indo-European and Dravidian linguistic groups has previously been reported [45]. STRUCTURE analysis (Figure S9) and PCA cluster plots (Figure S10) defines the single Indo-European isolated population (IE-E-IP1) as ‘Sino-Tibetan Indian’, with minimal European contribution, and the Dravidian population (DR-S-LP3) as an independent population cluster. The significant impact of European colonization within India is particularly evident in GC3, which includes in our analysis the large Indo-European and the most northerly of the southern Dravidian populations. Geographically, the GC3 region contributed the largest estimated total number of slaves during Dutch colonization of southern Africa. The D6-Coloured showed almost equal ancestral contributions defined in this study as ‘broadly Indian’ (21%) and ‘Sino-Tibetan’ (22%). Confirming an Indian ancestral contribution to the D6-Coloured, it is however not possible to distinguish Sino-Tibetan from Indonesian using the minimal merged dataset.

## Discussion

Although previous studies have considered the role of admixture in shaping the genetic diversity among southern Africans, in particular the Coloured, no study has assessed (i) the significance of these contributions, (ii) how this admixture has shaped or contributed to distinct population subgroups among southern Africans, or (iii) the possibility that southern Africans may be harboring ancient vestiges of a ‘lost’ or understudied source of genetic diversity. The extent of admixture within people today defined broadly as Khoesan complicates these analyses, further compounded by subject heterogeneity. We attempt to assess sources of admixture and heterogeneity and ultimately identify and characterize a Khoesan-representative population that displays little to no significant non-Khoesan ancestral contribution. Such a population we identify as the Ju/'hoan.

This study suggests that the Ju/'hoan form a unique ancestral population for the human lineage, distinct (i.e., most dissimilar) from all contemporary populations for which data is currently available, including other forager populations. Gender-specific analysis confirms genetic isolation of the Ju/'hoan from non-Khoesan populations, while autosomal analysis shows no significant non-Ju/'hoan ancestral contribution. While the rest of the world was driven into agriculture at the end of the Last Glacial Maximum [48], the Ju/'hoan appear to have maintained their hunter-gatherer based subsistence. Significant agricultural-driven genomic signatures were absent from the study subjects, while previously described functionally significant ancestral forager-based alleles were identified. One of the most interesting findings to emerge from our analysis of foraging versus agricultural genome profiles was a potential for an increased chemical dependency for tobacco. We observed heavy tobacco usage by all study participants, both male and female. Historical accounts include the successful use of tobacco as a means of trade or coercion of indigenous Khoesan by European settlers [49]. Anthropological observational studies suggest an unusual devotion of Ju/'hoan to master the difficult task of tobacco cultivation over food-based cultivation when minimal farming is adopted [50]. Our data therefore suggests that the Ju/'hoan have not had adequate time to adapt to selective pressure associated with the use of tobacco. The significance of genes associated with inflammatory, autoimmune or immune diseases, being significantly enriched between forager and agriculturalist requires further investigation. Coined ‘the harmless people’ [51], it may not be surprising that we found a greater representation of loci associated with mood-based disorders. Physical characteristics within the Ju/'hoan with possible links to enriched pathways include (i) maintaining both thermal and fluid homeostasis within desert climates, (ii) the need for rapid wound repair, and (iii) a possible state of semi-erection in males. The latter, a locally accepted trait, has been documented in Bushmen rock art [52] and reported as a defining characteristic [53].

Unlike the Ju/'hoan, the !Xun exhibit significant male-derived non-Khoesan African ancestral contribution to their gene pool. While autosomal marker analysis suggests roughly 20.5% non-Khoesan admixture, Y-chromosomal analysis suggests a possible East African Nilotic contribution, although extended autosomal substructure analysis suggests a proto-Bantu and Sandawe contributions while excluding for a Nilotic contribution. Evidence for Bantu migration into the northern Kalahari region of Namibia appears as early as the 7th century [54]. Bantu-Khoesan interaction is evident by the introduction of iron-based arrow tips and cooking utensils, as well as the use of cultivated tobacco by the Khoesan, and conversely the inclusion of clicks within the non-click languages of early Bantu immigrants, for example isiXhosa (the language of the amaXhosa). The possibility of a pre-Bantu, likely east African migration into the region requires further investigation. The Ju/'hoan-Yoruba differentiating AIMs defined two unique !Xun subgroups suggesting independent genomic prehistories. The Ju/'hoan-ancestral !Xun share on average 54.8% (range 43.6–67.5%) of their genomic heritage with contemporary Ju/'hoan, and include the Angolan !Xun from this study (Figure 4C). In contrast, we identify a new non-Ju/'hoan (range 0.9–5.3%) ancestral contribution to 50% of the !Xun, averaging 71.1% (range 65.9–76.9%) (Figure 4D). We suggest that the !Xun identifier as used today incorporates different Khoesan prehistories, one independent from contemporary Ju/'hoan. Interestingly, the non-Khoesan African contribution to the !Xun appears to be uniform with ancestral signatures shared by contemporary Bantu and Sandawe. Our data therefore suggests that these two independent !Xun lineages carry the same non-Khoesan African contributions. The amaXhosa Bantu carry an almost equal ancient ancestral Khoesan contribution, while AIMs analysis suggests that this contribution is largely non-Ju/'hoan. It is highly feasible to assume that the southward migration of the amaXhosa along the eastern coast would constitute differing Khoesan contribution from the more westerly located inland Ju/'hoan. This observation is further supported by the lack of L0k mtDNA representation within the amaXhosa. Further analysis would be required to determine the relationship between the Khoesan contribution to the amaXhosa and the ‘unknown’ !Xun lineage identified in this study.

While the !Xun and amaXhosa show evidence for historical admixture, inter-continental migrations to the region has led to the emergence of more recent admixture. Considering a highly variable non-Khoesan contribution to the #Khomani, the Coloued and Baster populations represent a complex admixture pattern that transverses both the earliest and the most recently diverged human lineages. Defining and tracing such significant ancestral contributions provides a unique model not only to track human expansion and prehistories, but also define gene regions undergoing selection [55]–[57] and recombination [58], [59]. The datasets presented in this study provide a unique resource for further genomic analyses. In the Ju/'hoan we speculate that the fraction of ROH has been lowered as a result of early divergence with other populations, while increased as a result of a smaller effective population size (*Ne*). Unlike cosmopolitan societies, the maintenance of population size is an essential survival mechanism for foragers. As a result of varied contribution of ancestrally distinct chromosomal segments, contemporary southern African populations would display admixture-based recombination, decreasing total ROH. The complex ‘Khoesan-African-Asian-European’ ancestral admixture fractions of the Baster and Coloured would be further impacted by gender-specific meiotic recombination rates [60]. The observation of gender biased ancestral contributions include a paternally-driven ‘African non-Khoesan’ contribution to the !Xun, maternally-driven ‘Khoesan’ contribution to the amaXhosa, and maternally-driven ‘Khoesan’ and paternally driven ‘non-African’ (likely European) contribution to the Baster and Coloured.

Although previous studies have looked at the ancestral contributions to the Coloured [17], [18], no studies have to date addressed complex admixture within the Basters. Emerging from a common historical background to the Coloured, the Baster population have since the late 1800 s distinguished themselves as independent from the Coloured, migrating to the now Baster nation of Rehoboth in Namibia [9]. In contrast to the Coloured we show the Baster population to carry the largest Khoesan-derived maternal contribution (91.7% compared to 64.3% in the Coloured) and the largest paternal European-derived contribution (93.3% compared to 71.8%), while autosomal marker analysis confirmed increased ‘Khoesan’ and ‘European’ contributions and decreased ‘Asian’ and ‘African non-Khoesan’ contributions. Geographic distribution of the ‘African non-Khoesan’ admixture fraction showed an increased contribution and significance from west to east (Baster, NC-, D6- to EC-Coloured, Figure S7), with significance of the Bantu-derived fraction (1.6%, 5.8%, 15.4% and 16.6%, respectively) based on nine ancestral fractions (Figure 2B) and mirroring Bantu population distributions (Statistics South Africa Census 2011 and Community Survey 2007, (http://www.statssa.gov.za)). The most significant ‘Asian’ contribution was found within persons who were residents of District Six. Previously a residential region of Cape Town, District Six was geographically located at the heart of the Dutch-East Indian slave trade [43], [47]. In this study we define an almost equal ‘broadly Indian’ and ‘Sino-Tibetan’ contribution to the D6-Coloured. Besides fixation for the dry earwax allele in the Han Chinese and Koreans, an elevated frequency (71%) has been reported for the Indian Dravidian inhabitants of Tamil Nadu (correlating to the DR-S-LP3 population from this study) [41]. Lack of this allele in our subjects alludes to a non-Dravidian Indian contribution which was further supported by non-contributing independent GC4 Dravidian subgroup substructure.

Since the submission of this paper, two publications have emerged that have addressed genomic variation within the southern African region we studied. The first assessed ∼500 K custom designed variants including study subjects described as Ju/'hoan and !Xun (!Xuun) and grouped together as Kx'a speakers [61]. Significant findings consistent with our analyses include ∼20% non-Khoesan contribution to the !Xun (after fixing non-Khoesan contribution to the Ju/'hoan at 6%), while confirming minimal admixture contribution within the Ju/'hoan. Additionally this study dates the !Xun African non-Khoesan-mixture time to around 450 years ago and implies an ancient genetic link between southern and Eastern Africa. Our observation for a predominance of the East African Nilotic (non-Bantu) E1b1b Y-chromosomal haplogroup within the !Xun may provide further confirmation for a southern-eastern link, although our autosomal analysis suggests that this link is more likely related to the Sandawe and not the Nilotic peoples. No ancestral link was observed between east Africans and the Ju/'hoan from our study. The second paper looked at ∼2.3 million variants including study subjects described as Ju/'hoan, !Xun, Coloured (Colesburg), Coloured (Wellington) and undefined South African Bantu-speakers [62]. Consistent with our findings and the first paper, this study depicts the Ju/'hoan as a relatively homogenous population, while depicting a non-Khoesan contribution to the !Xun. In contrast to both studies, we suggest additional !Xun substructure and present the notion of two distinct !Xun prehistories. Our assumption is that contemporary !Xun represent a unique ancestral Khoesan lineage with an ancient non-Khoesan African (predominantly Bantu) contribution, with one subgroup having shared an ancient genetic link with the Ju/'hoan while the other remained genetically isolated from the Ju/'hoan. Notably the second study reports a predominance of Angolan !Xun study representation, represented in our study by the Ju/'hoan-ancestral genetic link. Further between study confirmation includes the representation of a South Asian (Indian) contribution to the Coloured, in particular the Wellington-Coloured (approximately 60 miles from Cape Town and District Six) compared with the Colesburg-Coloured (approximately 500 miles from Cape Town and District Six), with minimal East Asian ancestral contribution. Unlike the Wellington-Coloured, however, no subject in this study presented with non-African ancestry (Bantu and/or Khoesan). A single individual from District Six lacked any observable Khoesan contribution. No distinction was made for the Southern Bantu included in the latter study, so no correlation could be made with regards to the amaXhosa. The availability of new southern African datasets will allow for a more comprehensive analysis of population substructure within the region.

This study demonstrates both ancient and recent admixture within southern Africans. Cautionary concerns include: (i) bias in current content arrays towards non-African populations will greatly impact inferences about diversity among southern Africans, while lack of rare allele representation would diminish an ability to separate southern African subpopulations, (ii) lack of an available common ancestral genome that truly represents the earliest modern humans results in biases in methods used to attain divergence times among populations, (iii) inferences regarding population structure and recent admixture events are currently based on analyses of data reflecting contemporary genetic variation between populations, which is still largely lacking for the region of Southern Africa, and (iv) this is confounded by lack of data for populations that may actually be extinct. Taking these cautionary observations into consideration, we present an analysis of a set of individuals that, to the best of our knowledge, most accurately defines a homogenous ancestral Khoesan contribution, the Ju/'hoan. Additional cultural differences that may have restricted interbreeding between our Ju/'hoan and local agro-pastoral groups include; economic distinction (those without and those with possessions), language (Khoesan versus Bantu), social practices (egalitarian versus patriarchal society), kinship (bilineal/patrilineal versus matrilineal), marital locality (matrilocal versus patrilocal), and marital practices (monogamy versus polygamy, and no bridal payment versus a bridal payment). While a recent study acknowledges western influences as a result of the establishment of a Ju/'hoan ‘reserve’ near Tsumkwe [62], for this reason we actively avoided recruitment within the immediate vicinity of Tsumkwe. In contrast to the Ju/'hoan, we describe not only a ‘African non-Khoesan’ (almost equal proto-Bantu and Sandawe) contribution to the !Xun, but define two distinct !Xun lineages, with, and largely without, a shared Ju/'hoan ancestry. Additionally we describe a new population with complex ancient and recently diverged genomic contribution, the Basters of Namibia. Sharing a history with the South African Coloured, population-defining genetic signatures include increased significance of Khoesan and European contribution with gender-specific bias to a maternal and paternal contribution, respectively. In contrast, while we confirm increased ‘African non-Khoesan’ (largely Bantu and to a lesser extent Sandawe) and ‘Asian’ (Indian and Indonesian) contribution to the Coloured, we demonstrate significant regional-based ancestral differences which would have important implications for gene mapping studies that rely on self-reported ancestry among Coloured and non-Coloured populations. As inter- and intra-continental migration increases globally, so will the impact of admixture on disease gene mapping studies. The dataset presented provides an opportunity to investigate the impact of arguably some of the most diverse genomic contributions within single population identifiers.

## Materials and Methods

### Ethics statement

The study was approved by the Ministry of Health and Social Services in Namibia, the human Research Ethics Committee at the University of Stellenbosch, South Africa (Project # N08/03/072), the Institutional Review Board Committee at the J. Craig Venter Institute (IRB# 2010-126) and previously the Human Research Ethics Committee at the University of New South Wales Australia (HREC# 08244). Consents were acquired either via verbal or written documentation with the understanding that the data generated will be made freely available to the scientific community as a collective. There are no known cultural limitations that would prohibit open access of the data.

### Population identifiers and sampling

#### Ju/'hoan and !Xun

The symbols represent dental (/), alveolar (!), palatal (≠), and the lateral (//) clicks used in the Khoesan languages. Although historically the Ju/'hoan (n = 19) and !Xun (n = 14) have had no collective name for themselves [63], the term Khoesan (over Khoisan based on the linguistic observation that the combination of *o+i* does not exist in the Khoekhoegowab language) is used in this study to represent southern African foragers. Academically, Khoesan more accurately reflects two unique cultural identities the hunter-gatherer (San) and herder-gatherer (Khoe), while locally ‘Bossiesman’ (Afrikaans for Bushmen) is the more accepted collective identifier. The Working Group of Indigenous Minorities within Southern Africa (WIMSA; http://www.wimsanet.org/) identifies 13 Bushmen or ‘San’ language groups, which include the Ju/'hoan and the !Xun (or !Kung). Representing Ju languages [64], the !Xun originate from Namibia or Angola, the latter relocated to Namibia are also self-identify as Vasekela (although less frequently used). Subjects were specifically recruited from remote areas within Namibia with avoidance of significant western developments and family units demonstrating extensive co-existence with non-Ju/'hoan or non-!Xun (as evident in the many reserve-like establishments). All subjects were extensively interviewed with regards to local history, family history, subsistence (current and past), regional movements, impact within the family unit from other Khoesan and non-Khoesan groups, anthropological-based practices, etc. For further information see http://www.jcvi.org/cms/research/projects/southern-african-genome-diversity-study/.

#### amaXhosa

The amaXhosa are a Southern Bantu people who speak isiXhosa from the Nguni classification. Bantu migrations are believed to have originated from north-western Cameroon/southern Nigeria around 5-3 thousand years ago [7], including the Southern Bantu [65]. Encounters with the Khoesan led not only to the incorporation of ‘click’ sounds in the isiXhosa non-Khoesan language [64], but also genetic contributions. Previous studies have reported mtDNA [14] and Y-chromosome [15] Khoesan contributing haplogroups within Southern Bantu speakers (specifically the amaXhosa, amaZulu, Basotho and Batswana). All study subjects (n = 15) self-identified via their maternal and paternal ethnic classification and all identified with ancestral roots within the Eastern Cape region of South Africa.

#### Coloured and Baster

Population admixture involving the Khoesan and additional founder populations started shortly after the arrival of the Dutch-East Indian Company in 1652 at the Cape of Good Hope (now Cape Town, South Africa). These early migrants included European settlers (predominantly Dutch, German and French, and later British) and slaves from the East (including India, Indonesia, and Sri Lanka), Madagascar, and coastal Africa (including Mozambique, Angola and Guinea) [9], [66]. The slave trade continued for almost two centuries and resulted in the founding of the Coloured people. The Basters, formerly part of the original pool of Cape admixed individuals, traveled north towards then South West Africa (now Namibia) and settled in the town of Rehoboth where in 1870 they established themselves as an independent population. In this study, place of birth was used for regional subgroup classification and candidate selection. All the Basters were from Rehoboth (n = 30), while the Coloured (n = 25) were geographically classified as District Six (D6, n = 8), Eastern Cape (EC, n = 7) or Northern Cape (NC, n = 10). All study subjects self-identified via both their maternal and paternal heritage.

#### Use of “controversial” population identifiers

The terms Bantu and Coloured have different meanings to different people, the latter exasperated by the use of a parallel term within the United States (Colored). Until 1991 South African law divided the country into four official ethnic groups, defined as ‘Black (or Bantu)’, ‘Coloured’, ‘Indian’ and White’. Although may South Africans still use these population identifiers, these classifications undoubtedly have negative connotations for certain people. The use of Bantu or Southern Bantu in this paper refers to the widely accepted linguistic identifier. The continued use of the term Coloured in this study was based on a survey of 521 persons who self-identified as Coloured (91.2%), including the use of terms ‘South African Coloured’ and ‘Cape Coloured’, while the remaining 8.8% referred to themselves as Admixed/mixed ancestry or Other. Interviews were unprompted and performed by one of the Coloured co-authors. Of the 51.8 million South Africans registered in the 2011 census, 4.62 million (11.2%) self-identified as ‘Coloured’ (http://www.statssa.gov.za/census2011/). Based on our survey, the 2011 census and in agreement with the two self-identified Coloured co-authors, we conclude that Coloured is still the most widely recognized population-specific identifier within the community. The use of the term Baster (or Rehoboth Baster) is regarded with immense pride within the community, establishing themselves as a Republic with a national flag [67].

### Genotyping and QC

Genomic DNA was isolated from whole blood using the QIAamp DNA Blood Mini kit or the FlexiGene DNA kit (QIAGEN) and quantified on the NanoDrop spectrophotometer (Thermo Scientific). A total of 105 subjects were genotyped using the Illumina HumanOmni1-Quad Beadchips. The Illumina GenomeStudio software (version1.7.4) was used for the data analysis with a GenTrain score of 0.5 as the minimum for inclusion. Excluding indels, mtDNA, Y-chromosome markers and no-calls resulted in 927,298 variants. Two subjects (both Ju/'hoan) were excluded as a result of likely first cousin relatedness based on the estimation of the probable number of shared alleles at any given marker, identity by descent (IBD) values of 0.4351 and 0.4129. Genotype calls have been made available without restrictions at http://www.jcvi.org/cms/research/projects/southern-african-genome-diversity-study/ for the complete dataset of 103 subjects according to population identifiers.

### Gender-specific analysis

MT-haplogroup specific markers (Table S1) were identified using the phylogenetic tree (www.phylotree.org) build 13 [68], amplified and Sanger sequenced. Briefly, PCR was performed using the FastStart Taq system (Roche), product cleanup using the ExoSap method and Sanger Sequencing using the BigDye terminator cycling kit and the ABI 3100 Genetic Analyzer (Applied Biosystems). Paternally derived Y-chromosome haplogroups were assessed from a total of 1,283 Y-chromosome markers represented in the Illumina Human Omni1-Quad array content. Markers were identified from the Y-Chromosome Consortium (YCC) 2008 nomenclature [69]. E1b1b-specific marker M215 (rs2032654) was determined by amplicon-specific Sanger sequencing. All primer information and amplification conditions are available within Table S14.

### Genome-wide autosomal analysis

#### Merged datasets and LD-pruning

The sample base (n = 103, 5 populations) was merged with two publically available datasets namely, (i) the Illumina iControl database (http://www.illumina.com/) including Human Omni1-quad data, specifically Yoruba (n = 90), European (n = 175) and Han Chinese (n = 44), and allowing for an autosomal overlap of 927,298 autosomal markers and (ii) the published dataset of Henn et al., 2011 [4] containing 53,811 autosomal markers (www-evo.stanford.edu/pubs.html) and including the #Khomani (n = 31), Biaka (n = 22), Mbuti (n = 13), Hadza (n = 17), Sandawe (n = 28), Maasai (n = 30) and Luhya (n = 36). Total autosomal overlap after merging all three datasets was 33,207 variants and 15 population identifiers. All merged variants underwent strand designations checks. Total number of individuals interrogated was dependent on the analysis required. Thinning the marker set for LD was based either on the analysis to be performed not explicitly taking LD into consideration and/or the time required to run the analysis. For LD-pruning we removed variants with an R^2^ value greater than 0.1 or less stringent 0.2 with any other variant within a 50-variant sliding window, advanced by 5 variants at a time. The total number of variants interrogated for LD-pruned datasets are highlighted throughout the main text.

#### Merged Asian-specific datasets

Two non-platform-matched ‘Asian’ derived datasets were merged with our data to further define the ‘Asian’ ancestral contribution, in particular the Coloured and District Six Coloured. These include (i) published Affymetrix SNP6.0 data for 20 Indonesians representing 4 population identifiers, specifically Makassar, Bugi, Javanese and Batak Toba [17], and (ii) Affymetrix 50K genotype data supplied by the Indian Genome Variation Consortium for 179 Indians from 10 populations, selected based on geographical distribution, and as previously published [44]–[46]. Indian subjects were classified linguistically as Tibeto-Burman (TB), Austro-Asiatic (AA), Indo-European (IE) or Dravidian (DR), geographically as north (N), northeast (NE), central (C), east (E), west (W) or south (S), or by caste/religious group/tribe size as a large population (LP) or an isolated population (IP) [46]. Merging with the Indian dataset generated 9,317 overlapping autosomal markers, while including the Indonesian data generated 3,725 overlapping autosomal markers. All merged variants underwent strand designations checks.

#### Analytical methods

Multiple analytical methods were used in this study. Each method is described in brief. PCA was performed using the PCA tool in the HelixTree module of the ‘SNP and Variation Suite’ in the SVS 7.5 software available from Golden Helix (http://www.goldenhelix.com/), considering the first two eigenvectors. STRUCTURE 2.3.3 analyses [24] was performed using 5000 burn-in iterations, followed by 10000 iterations for the merged Illumina dataset of 33,207 markers (assuming K = 2 to K = 7 population clusters) and 10000 burn-in iterations, followed by 20000 iterations using the Ju/'hoan-Yoruba AIM-dataset of 2,687 markers (assuming K = 2 and K = 3) and the Indian merged dataset of 9,317 markers (assuming K = 2 to K = 5). A minimum of five runs was performed per ancestral estimation. GAMOVA was performed as previously described [25], allowing for the assessment of the degree to which genome-wide allele sharing among a set of individuals can be explained on the basis of population labels assigned to each individual. Circular and linear 100 NJ trees were created using ‘neighbor’ within the PHYLIP package (http://cmgm.stanford.edu/phylip/). The trees were rooted to the Chimpanzee (*Pan troglodytes*) genome (version PanTro2; http://genome.ucsc.edu). A single circular tree is represented. Determining the fraction of ROH using SVS 7.5, we first removed from the large 928,799 merged dataset markers with a minor allele frequency (MAF) less than 5% and markers with a Hardy Weinberg Equilibrium (HWE) P value less than 0.0001, leaving a total number of 716,734 markers (367 individuals). Parameters included a minimum run length of 500 kb with a minimum number of 25 variants and runs containing no more than 1 heterozygote and up to 5 missing genotypes. The minimum number of samples to contain a run was set to 1 with the maximum gap of 100 kb between variants and a minimum density of 1 variant per 50 kb in a run. The supervised mode of ADMIXTURE v1.22 [43] was used to calculate Fst values. Identity by state (IBS) was calculated using PLINK v1.07 software [70]. An assumption of six ancestral populations was used to determine significance of admixture fractions using ADMIXTURE. The distribution of estimated admixture fractions are shown using boxplots (Figure 4A), with the dashed line indicating significant overall ancestral contributions based on each individual from each of the populations. A value of 1.0 therefore implies that all individuals representing the admixed population have the presence of the corresponding ancestral contribution.

### Adaptation-associated and/or ancestry informative marker analysis

#### Marker-specific analysis

Markers specifically associated with human adaptations that further define population ancestry were identified from the literature and include; (i) the African-specific Duffy antigen receptor gene (*DARC*) promoter variant rs2814778 −46 A>G (associated with malaria resistance as a result of agricultural practices) [28], (ii) the pancreatic lipase-related protein 2 (*PLRP2*) nonsynonymous variant rs4751995 G>A, Trp358X (associated with an adaptation to grain cultivation) [29], (iii) three nonsynonymous variants, rs713598 C>G (Pro49Ala), rs1726866 C>T (Ala262Val), and rs10246939 C>T (Val296Ile), in the taste receptor, type 2, member 38 (*TAS2R38*) gene (associated with a loss in the ancestral taster haplotype which enables forager societies to detect dietary toxins) [30], (iv) four nonsynonymous variants within the arylamine N-acetyltransferase 2 (*NAT2*) gene, rs1801279 G>A (Arg64Gln), rs1801280 T>C (Ile114Thr), rs1799930 G>A (Arg197Gln) and rs1799931 G>A (Gly286Glu) (associated with a loss in the ancestral foraging required ability to rapidly eradicate dangerous dietary toxins) [31], (v) truncating variant rs6661174 C>T (Gln472X) in the flavin-containing monooxygenase 2 (*FMO2*) gene (associated with increased industrial-based toxicity and therefore selective disadvantage in western societies) [32], [33], (vi) the nonsynonymous variant within the catechol-O-methyltransferase (*COMT*) gene, rs4680 G>A, Val158Met (associated with loss of the ancestral ability to instantly react to adverse stimuli, a requirement for forager survival) [35], [36], (vii) the rs17822931 AA-genotype in the ATP-binding cassette transporter sub-family C member 11 (*ABCC11*) gene (associated with dry earwax in East Asians) [41], and (viii) the rs671 A-allele in the Aldehyde dehydrogenase 2 (*ALDH*2) gene (associated with alcohol induced facial flushing in Asians) [42]. Genotyping was performed using a custom designed Golden-Gate genotyping assay and the Veracode technology of Illumina (Illumina Inc., San Diego, CA, USA). The Illumina BeadXpress Reader was used for genotype identification and analyzed using the Illumina Beadstudio software (version 3.2.32).

#### Ancestry informative markers (AIMs)

The Manhatton plot analysis function in SVS 7.5 was used to generate the Ju/'hoan-Yoruba AIMs after LD pruning using parameters 50 (sliding window), 5 (variant advance) and 0.1 (R^2^) allowing for the analysis of 70,733 autosomal markers from 19 Ju/'hoan and 90 Yoruba. The variables (phenotypes) selection was based on population identifiers, namely Ju/'hoan versus Yoruba, allowing for the identification of alleles at significantly represented between the populations. A conservative threshold was set at −log10 P>5 for significance resulting in 2,687 AIMs. STRUCTURE analysis was performed on a reduced subset of Yoruba (n = 20) to facilitate visualization of the data generated. Further inclusion of all 90 Yoruba did not impact the overall ancestral contributions (data not shown). The MetaCore suite from GeneGo (http://www.genego.com/) was used to rank the list of generated AIMs based on gene ontology.

## Supporting Information

Figure S1 Geographical locations of Khoesan sampling sites included in this study. Study subjects were recruited from 11 locations across the north-western geographical region of Namibia and classified as either Ju/'hoan (orange, n = 21) or !Xun (green, n = 14). IBD allele sharing resulted in the exclusion of two Ju/'hoan from our study as a result of possible relatedness (total n = 19).
(PDF)

Figure S2 Gender-specific ancestral haplotype contributions to the regionally defined South African populations, amaXhosa, Coloured and Basters. Marker analysis with contributions from ancestral populations shown as percentages for (A) ‘African Khoesan’ (L0d), ‘African non-Khoesan’ (L, not L0d/L0k) and ‘non-African’ (N/M) mtDNA haplogroups and (B) ‘African non-Khoesan’ (E) and ‘non-African’ (R/I/G/N/O/J) Y-chromosome haplogroups. Distribution correlates with geographical distribution around the southern coast of Africa from the northwesterly Basters, to the westerly NC-Coloured, southerly D6-Coloured and the easterly located EC-Coloured and amaXhosa. This west to east distribution is correlated with a decrease in maternal ‘Khoesan’ and increase in ‘African non-Khoesan’ contribution, and a decrease in paternal ‘non-African’ and increase in ‘African non-Khoesan’ contribution. ‘Asian’ derived maternal contributions are highest at the geographical location of colonization and slave trade, at the most southerly region represented by the District Six Coloured.
(PDF)

Figure S3 Relatedness of the Ju/'hoan (n = 19) and !Xun (n = 14) in our study to published data for African populations defined as hunter-gatherer/click-speakers. PCA plot for 31,271 autosomal markers for 144 individuals from 7 populations defined as hunter-gatherer and/or click-speaking from within Africa. The Tanzanian and Pygmy lineages form independent population clusters to the Southern African Khoesan populations. The Ju/'hoan and !Xun form tight population clusters (excluding the single !Xun individual NF2). The #Khomani show a diversity in ancestral contributions, likely as a result of recent admixture.
(PDF)

Figure S4 Functionally relevant alleles/haplotypes associated with forager versus non-forager societies and their distribution within the Ju/'hoan and !Xun foragers and the agriculturalist amaXhosa. (A) The Duffy antigen receptor gene promoter variant (G-allele) associated with protection against malaria infection in Africa, (B) the pancreatic lipase-related protein 2 nonsynonymous variant (A-allele) associated with a diet rich in grains, (C) the taste receptor type 2 member 38 ‘taster’ (PAV) or ‘non-taster’ (AVI) haplotype associated with an ability or inability to taste toxins, (D) the arylamine N-acetyltransferase 2 ‘fast’ acetylation (G-T-G-G) haplotype associated with toxin irradication, (E) the flavin-containing monooxygenase 2 truncating inactive variant (T-allele) is absent in our forager groups, while the active full length C-allele is present enabling metablization of foreign toxins, and (F) the catechol-O-methyltransferase nonsynonymous variant (A-allele) decreased enzymatic activity associated with increase in anxiety-related conditions, while the ancestral G-allele is associated with a need for instinctive behavior. Note the English spelling with omits the prefix has been used to identify the amaXhosa.
(PDF)

Figure S5 Manhattan plot analysis for Ancestry Informative Marker (AIM) identification. A total of 70,733 LD-pruned Illumina Omni-quad 1M array markers were used to identify 2687 AIMs distinguishing the Ju/'hoan from our study (n = 19) to the Illumina iControl database Yoruba (n = 90), defined as a significant allele frequency difference between these population identifiers at −log10 P>5.
(PDF)

Figure S6 Significantly enriched biologically relevant pathways of genes that distinguish Ju/'hoan and Yoruba. Significant ancestry informative markers that distinguish the hunter-gatherer Ju/'hoan in our study from a representative of agriculturally-defined Yoruba, defined by a −log P value greater than 5, that are most significantly represented within genes grouped within biologically relevant pathways.
(PDF)

Figure S7 Regional distribution of the study sample and autosomal ancestral contributions. While the ‘African non-Khoesan’ (Bantu and Sandawe) contribution increases from west to east, the ‘European’ contribution decreases. Although maternal ‘Khoesan’ contributions decrease from west to east, autosomal analyses, although showing highest frequencies in the western populations, shows decreased frequency of contributions at the southern point and an inverse ‘Asian’ contribution.
(PDF)

Figure S8 Ju/'hoan and Yoruba ancestral contributions to the !Xun and amaXhosa. STRUCTURE analysis using the 2687 Ju/'hoan-Yoruba AIMs assuming two (estimated log likelihood of probability of data −160813.9) and three (−161042.6) population clusters (10000 burn-ins and 20000 iterations). K = 3 is also presented in the main text (Figure 4A).
(PDF)

Figure S9 Structure analysis for 9,317 LD pruned autosomal SNPs using K = 2 to K = 5 for ancestral populations with 5000 burn-in iterations and 10000 iterations. Indian populations listed as 1 to 10 in accordance with Figure 5B.
(PDF)

Figure S10 Principal component analysis (PCA) including 9,317 LD pruned autosomal markers for (A) D6 Coloured, Han Chinese, European and 10 Indian populations (B) D6 Coloured, Han Chinese and five Indian populations (GC1 and GC2) that were not impacted by colonization (C) D6 Coloured, European and five Indian populations (GC3 and GC4) that were impacted by colonization.
(PDF)

Table S1 Mitochondrial DNA haplogroups screened.
(PDF)

Table S2 Y-chromosomal haplogroup and subclade representation within the Baster and Coloured sample from our study.
(PDF)

Table S3 Multiple regression GAMOVA defines the contribution of variation explained by population identifiers.
(PDF)

Table S4 Fixed Ju/'hoan-specific alleles. Of the 2,687 ancestry informative Ju/'hoan-Yoruba markers (AIMs), 32 were found to be fixed in the Ju/'hoan (n = 19), 16 occurring in gene regions of potential functional relevance. Of the 16 fixed Ju/'hoan alleles, 13 are defined as ancestral against the *Pan* (Chimp) genome.
(PDF)

Table S5 Alleles with a highly significant −log10P>14 Ju/'hoan versus Yoruba population-specific association.
(PDF)

Table S6 Of the 2687 Ju/'hoan versus Yoruba AIMs (−log10P>5) 33 are nonsynonymous variants and are ordered in descending order from most significant.
(PDF)

Table S7 Ju/'hoan versus Yoruba differentiating AIMs located within genes ranked according to significance of genes enriched for biological processes.
(PDF)

Table S8 Ju/'hoan versus Yoruba differentiating AIMs located within genes ranked according to significance of genes enriched for molecular functions.
(PDF)

Table S9 Ju/'hoan versus Yoruba differentiating AIMs located within genes ranked according to significance of genes enriched for cellular localization.
(PDF)

Table S10 Ju/'hoan versus Yoruba differentiating AIMs located within genes ranked according to significance of genes enriched for disease associations.
(PDF)

Table S11 Allele sharing distances within (gray blocks) and between populations using identity-by-state (IBS) and including Fst distance values.
(PDF)

Table S12 Contribution of four ancestral populations to the amaXhosa, Baster and Coloured populations displayed as a percentage for both the average and range distributions.
(PDF)

Table S13 Significance of ancestral contributions within southern Africans from our study.
(PDF)

Table S14 Primer sequences and amplification conditions for gender-specific markers.
(PDF)
