# Supplementary material for: Complex Patterns of Genomic Admixture within Southern Africa
Source: PLoS Genet. 2013 Mar 14;9(3):e1003309. doi: 10.1371/journal.pgen.1003309 (PMC3597481; doi:10.1371/journal.pgen.1003309)
Supplement: Figure S1 — Geographical locations of Khoesan sampling sites included in this study. Study subjects were recruited from 11 locations across the north-western geographical region of Namibia and classified as either Ju/'hoan (orange, n = 21) or !Xun (green, n = 14). IBD allele sharing resulted in the exclusion of two Ju/'hoan from our study as a result of possible relatedness (total n = 19). (PDF) [file pgen.1003309.s001.pdf]

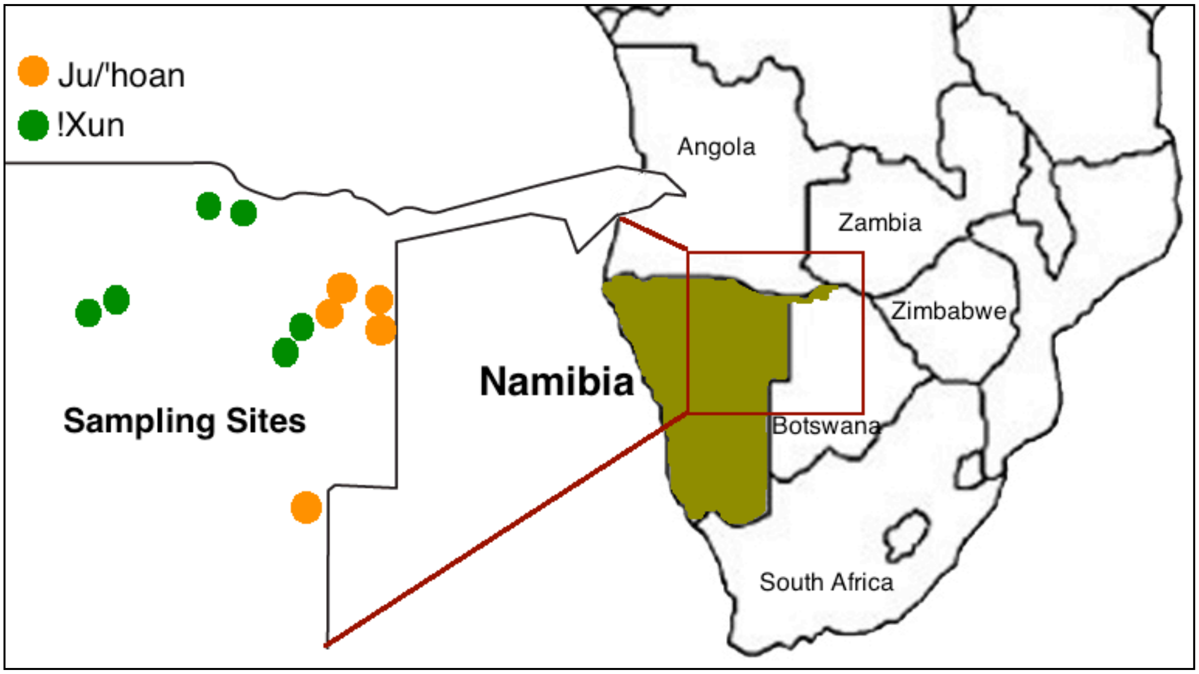

Figure S1. Geographical locations of Khoesan sampling sites included in this study. Study subjects were recruited from 11 locations across the north-western geographical region of Namibia and classified as either Ju/'hoan (orange, n=21) or !Xun (green, n=14). IBD allele sharing resulted in the exclusion of two Ju/'hoan from our study as a result of possible relatedness (total n=19).
