# Supplementary material for: Complex Patterns of Genomic Admixture within Southern Africa
Source: PLoS Genet. 2013 Mar 14;9(3):e1003309. doi: 10.1371/journal.pgen.1003309 (PMC3597481; doi:10.1371/journal.pgen.1003309)
Supplement: Figure S2 — Gender-specific ancestral haplotype contributions to the regionally defined South African populations, amaXhosa, Coloured and Basters. Marker analysis with contributions from ancestral populations shown as percentages for (A) ‘African Khoesan’ (L0d), ‘African non-Khoesan’ (L, not L0d/L0k) and ‘non-African’ (N/M) mtDNA haplogroups and (B) ‘African non-Khoesan’ (E) and ‘non-African’ (R/I/G/N/O/J) Y-chromosome haplogroups. Distribution correlates with geographical distribution around the southern coast of Africa from the northwesterly Basters, to the westerly NC-Coloured, southerly D6-Coloured and the easterly located EC-Coloured and amaXhosa. This west to east distribution is correlated with a decrease in maternal ‘Khoesan’ and increase in ‘African non-Khoesan’ contribution, and a decrease in paternal ‘non-African’ and increase in ‘African non-Khoesan’ contribution. ‘Asian’ derived maternal contributions are highest at the geographical location of colonization and slave trade, at the most southerly region represented by the District Six Coloured. (PDF) [file pgen.1003309.s002.pdf]

A **Regional Mitochondrial Haplotype Contributions**

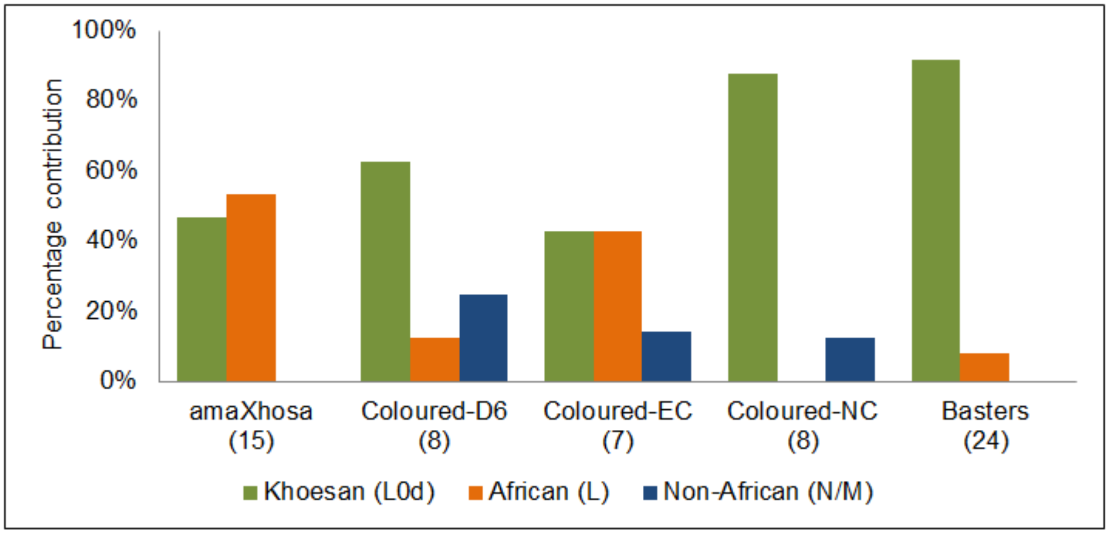

B **Regional Y-Chromosome Haplotype Contributions**

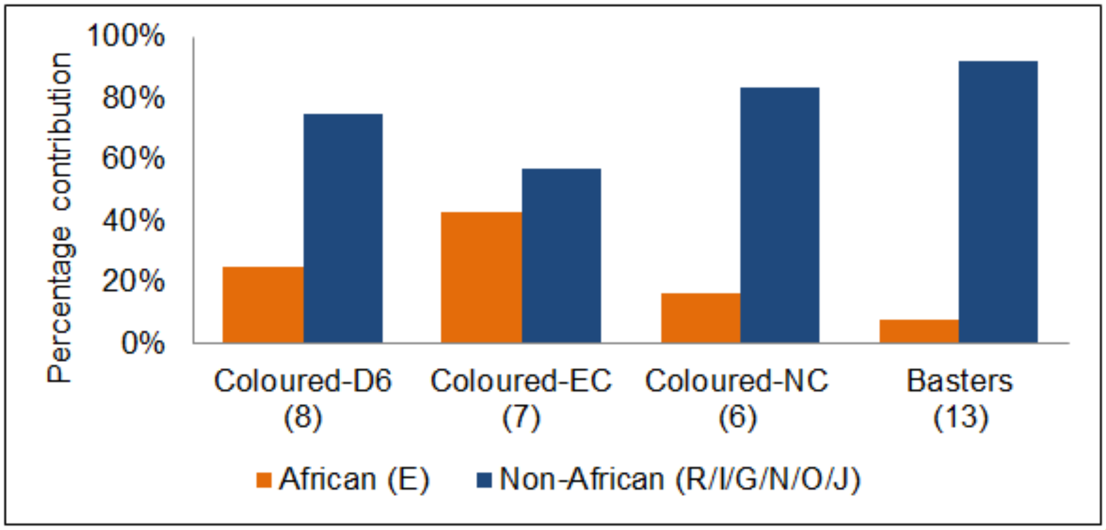

Figure S2. Gender-specific ancestral haplotype contributions to the regionally defined South African populations, amaXhosa, Coloured and Basters. Marker analysis with contributions from ancestral populations shown as percentages for **(A)** 'African Khoesan' (L0d), 'African non-Khoesan' (L, not L0d/L0k) and 'non-African' (N/M) mtDNA haplogroups and **(B)** **'**African non-Khoesan' (E) and 'non-African' (R/I/G/N/O/J) Y-chromosome haplogroups. Distribution correlates with geographical distribution around the southern coast of Africa from the northwesterly Basters, to the westerly NC-Coloured, southerly D6-Coloured and the easterly located EC-Coloured and amaXhosa. This west to east distribution is correlated with a decrease in maternal 'Khoesan' and increase in 'African non-Khoesan' contribution, and a decrease in paternal 'non-African' and increase in 'African non-Khoesan' contribution. 'Asian' derived maternal contributions are highest at the geographical location of colonization and slave trade, at the most southerly region represented by the District Six Coloured.
