# Supplementary material for: Complex Patterns of Genomic Admixture within Southern Africa
Source: PLoS Genet. 2013 Mar 14;9(3):e1003309. doi: 10.1371/journal.pgen.1003309 (PMC3597481; doi:10.1371/journal.pgen.1003309)
Supplement: Figure S3 — Relatedness of the Ju/'hoan (n = 19) and !Xun (n = 14) in our study to published data for African populations defined as hunter-gatherer/click-speakers. PCA plot for 31,271 autosomal markers for 144 individuals from 7 populations defined as hunter-gatherer and/or click-speaking from within Africa. The Tanzanian and Pygmy lineages form independent population clusters to the Southern African Khoesan populations. The Ju/'hoan and !Xun form tight population clusters (excluding the single !Xun individual NF2). The #Khomani show a diversity in ancestral contributions, likely as a result of recent admixture. (PDF) [file pgen.1003309.s003.pdf]

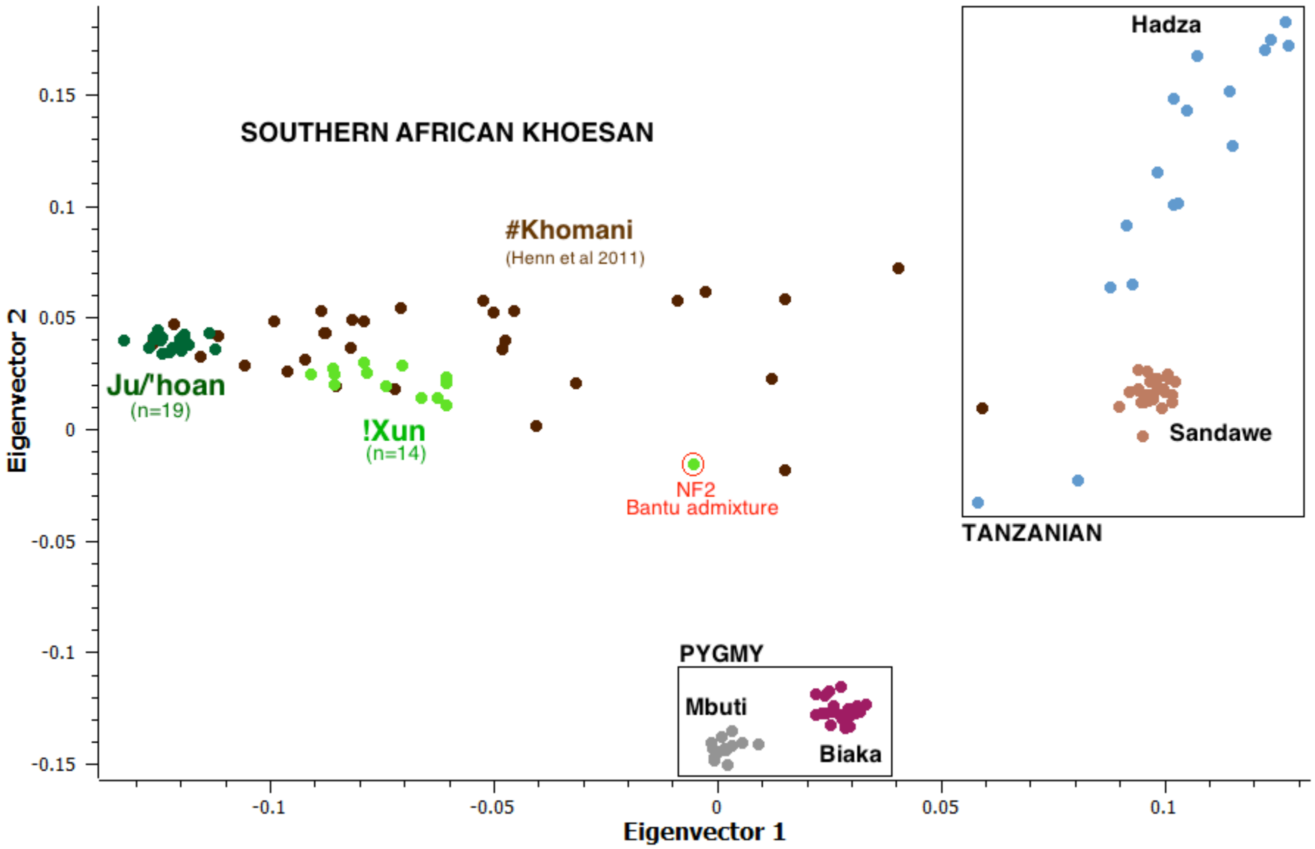

Figure S3. Relatedness of the Ju/'hoan (n=19) and !Xun (n=14) in our study to published data for African populations defined as hunter-gatherer/click-speakers. PCA plot for 31,271 autosomal markers for 144 individuals from 7 populations defined as hunter-gatherer and/or click-speaking from within Africa. The Tanzanian and Pygmy lineages form independent population clusters to the Southern African Khoesan populations. The Ju/'hoan and !Xun form tight population clusters (excluding the single !Xun individual NF2). The #Khomani show a diversity in ancestral contributions, likely as a result of recent admixture.
