# Supplementary material for: Complex Patterns of Genomic Admixture within Southern Africa
Source: PLoS Genet. 2013 Mar 14;9(3):e1003309. doi: 10.1371/journal.pgen.1003309 (PMC3597481; doi:10.1371/journal.pgen.1003309)
Supplement: Figure S4 — Functionally relevant alleles/haplotypes associated with forager versus non-forager societies and their distribution within the Ju/'hoan and !Xun foragers and the agriculturalist amaXhosa. (A) The Duffy antigen receptor gene promoter variant (G-allele) associated with protection against malaria infection in Africa, (B) the pancreatic lipase-related protein 2 nonsynonymous variant (A-allele) associated with a diet rich in grains, (C) the taste receptor type 2 member 38 ‘taster’ (PAV) or ‘non-taster’ (AVI) haplotype associated with an ability or inability to taste toxins, (D) the arylamine N-acetyltransferase 2 ‘fast’ acetylation (G-T-G-G) haplotype associated with toxin irradication, (E) the flavin-containing monooxygenase 2 truncating inactive variant (T-allele) is absent in our forager groups, while the active full length C-allele is present enabling metablization of foreign toxins, and (F) the catechol-O-methyltransferase nonsynonymous variant (A-allele) decreased enzymatic activity associated with increase in anxiety-related conditions, while the ancestral G-allele is associated with a need for instinctive behavior. Note the English spelling with omits the prefix has been used to identify the amaXhosa. (PDF) [file pgen.1003309.s004.pdf]

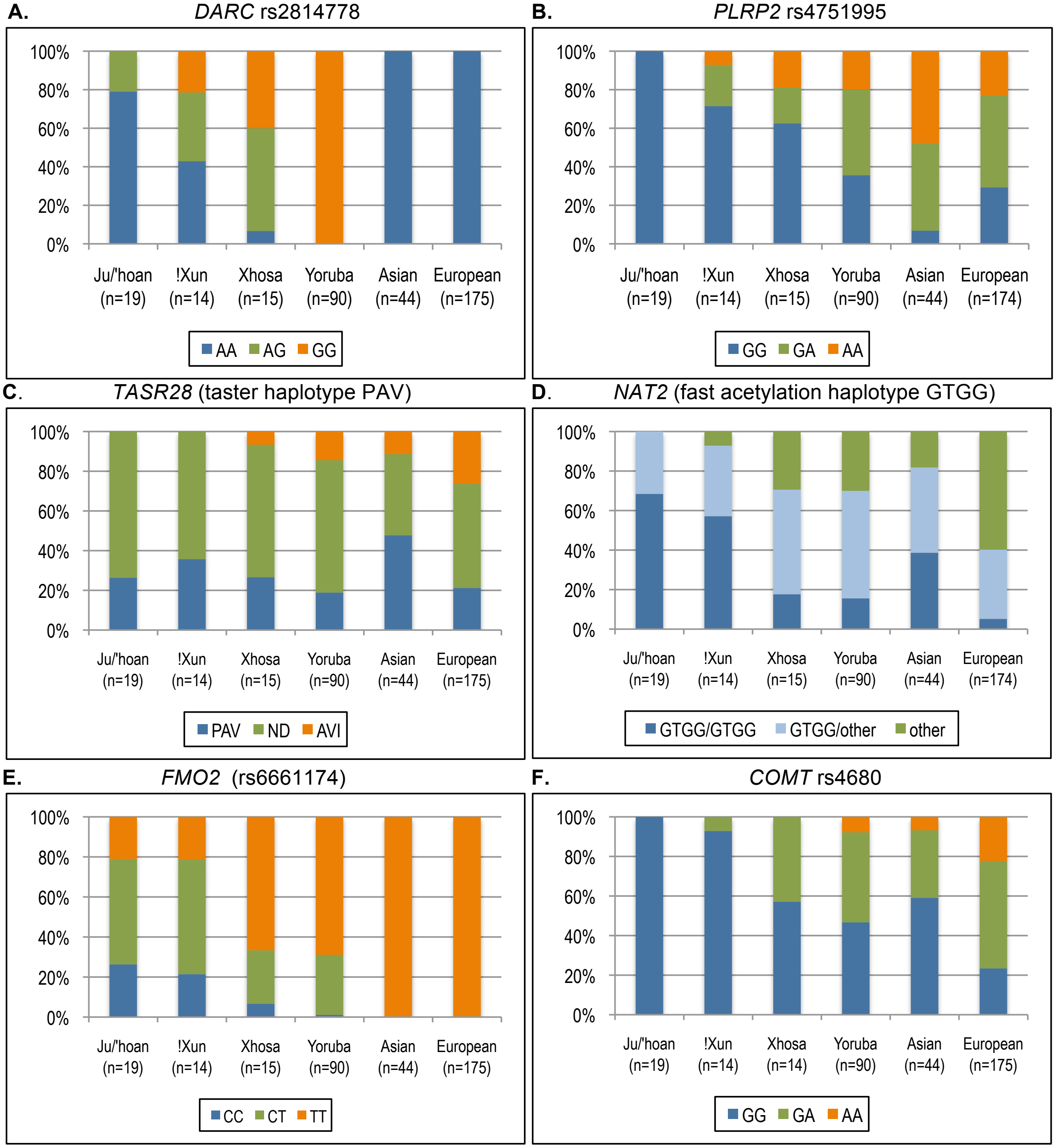

Blue: ancestral allele; Orange: derived allele; Green: heterozygous

Figure S4. Functionally relevant alleles / haplotypes associated with forager versus non-forager societies and their distribution within the Ju/'hoan and !Xun foragers and the agriculturalist amaXhosa. (A) The Duffy antigen receptor gene promoter variant (G-allele) associated with protection against malaria infection in Africa, (B) the pancreatic lipase-related protein 2 nonsynonymous variant (A-allele) associated with a diet rich in grains, (C) the taste receptor type 2 member 38 'taster' (PAV) or 'non-taster' (AVI) haplotype associated with an ability or inability to taste toxins, (D) the arylamine N-acetyltransferase 2 'fast' acetylation (G-T-G-G) haplotype associated with toxin irradication, (E) the flavin-containing monooxygenase 2 truncating inactive variant (T-allele) is absent in our forager groups, while the active full length C-allele is present enabling metablization of foreign toxins, and (F) the catechol-O-methyltransferase nonsynonymous variant (A-allele) decreased enzymatic activity associated with increase in anxiety-related conditions, while the ancestral G-allele is associated with a need for instinctive behavior. Note the English spelling with omits the prefix has been used to identify the amaXhosa.
