# Supplementary material for: Complex Patterns of Genomic Admixture within Southern Africa
Source: PLoS Genet. 2013 Mar 14;9(3):e1003309. doi: 10.1371/journal.pgen.1003309 (PMC3597481; doi:10.1371/journal.pgen.1003309)
Supplement: Figure S5 — Manhattan plot analysis for Ancestry Informative Marker (AIM) identification. A total of 70,733 LD-pruned Illumina Omni-quad 1M array markers were used to identify 2687 AIMs distinguishing the Ju/'hoan from our study (n = 19) to the Illumina iControl database Yoruba (n = 90), defined as a significant allele frequency difference between these population identifiers at −log10 P>5. (PDF) [file pgen.1003309.s005.pdf]

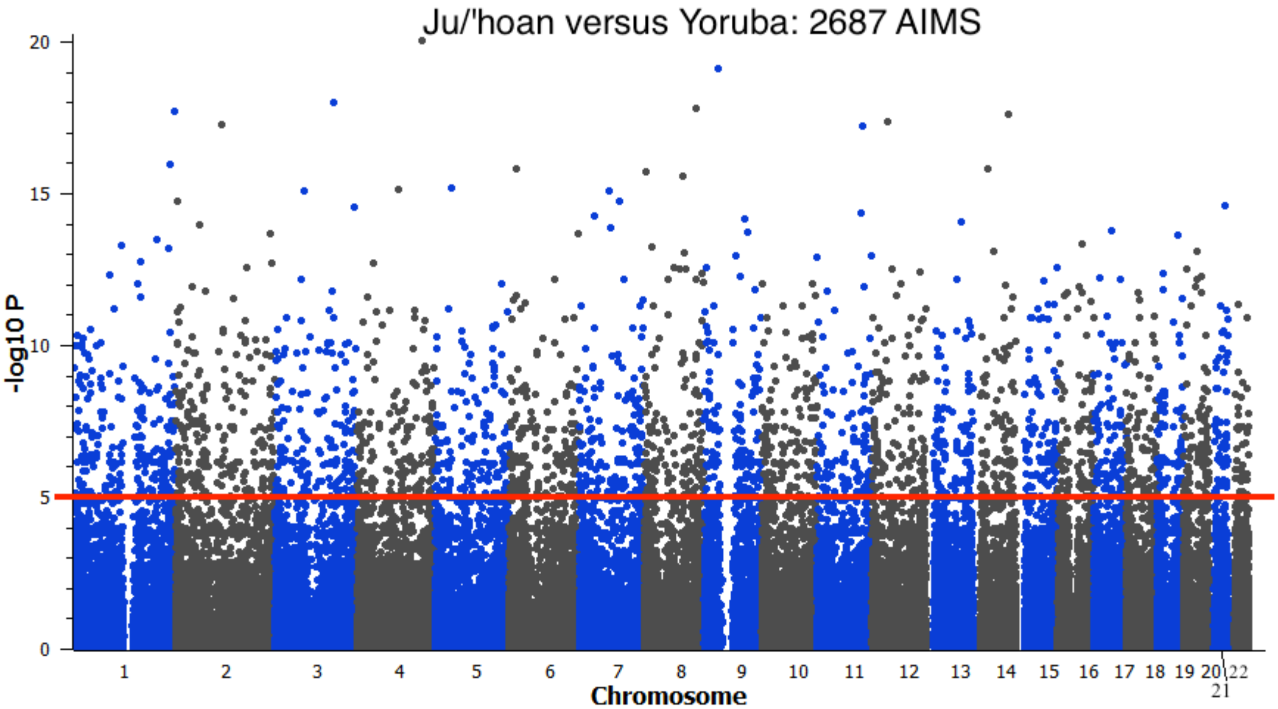

Figure S5. Manhattan plot analysis for Ancestry Informative Marker (AIM) identification. A total of 70,733 LD-pruned Illumina Omni-quad 1M array markers were used to identify 2687 AIMs distinguishing the Ju/'hoan from our study (n=19) to the Illumina iControl database Yoruba (n=90), defined as a significant allele frequency difference between these population identifiers at –log10 P>5.
