# Supplementary material for: Complex Patterns of Genomic Admixture within Southern Africa
Source: PLoS Genet. 2013 Mar 14;9(3):e1003309. doi: 10.1371/journal.pgen.1003309 (PMC3597481; doi:10.1371/journal.pgen.1003309)
Supplement: Figure S6 — Significantly enriched biologically relevant pathways of genes that distinguish Ju/'hoan and Yoruba. Significant ancestry informative markers that distinguish the hunter-gatherer Ju/'hoan in our study from a representative of agriculturally-defined Yoruba, defined by a −log P value greater than 5, that are most significantly represented within genes grouped within biologically relevant pathways. (PDF) [file pgen.1003309.s006.pdf]

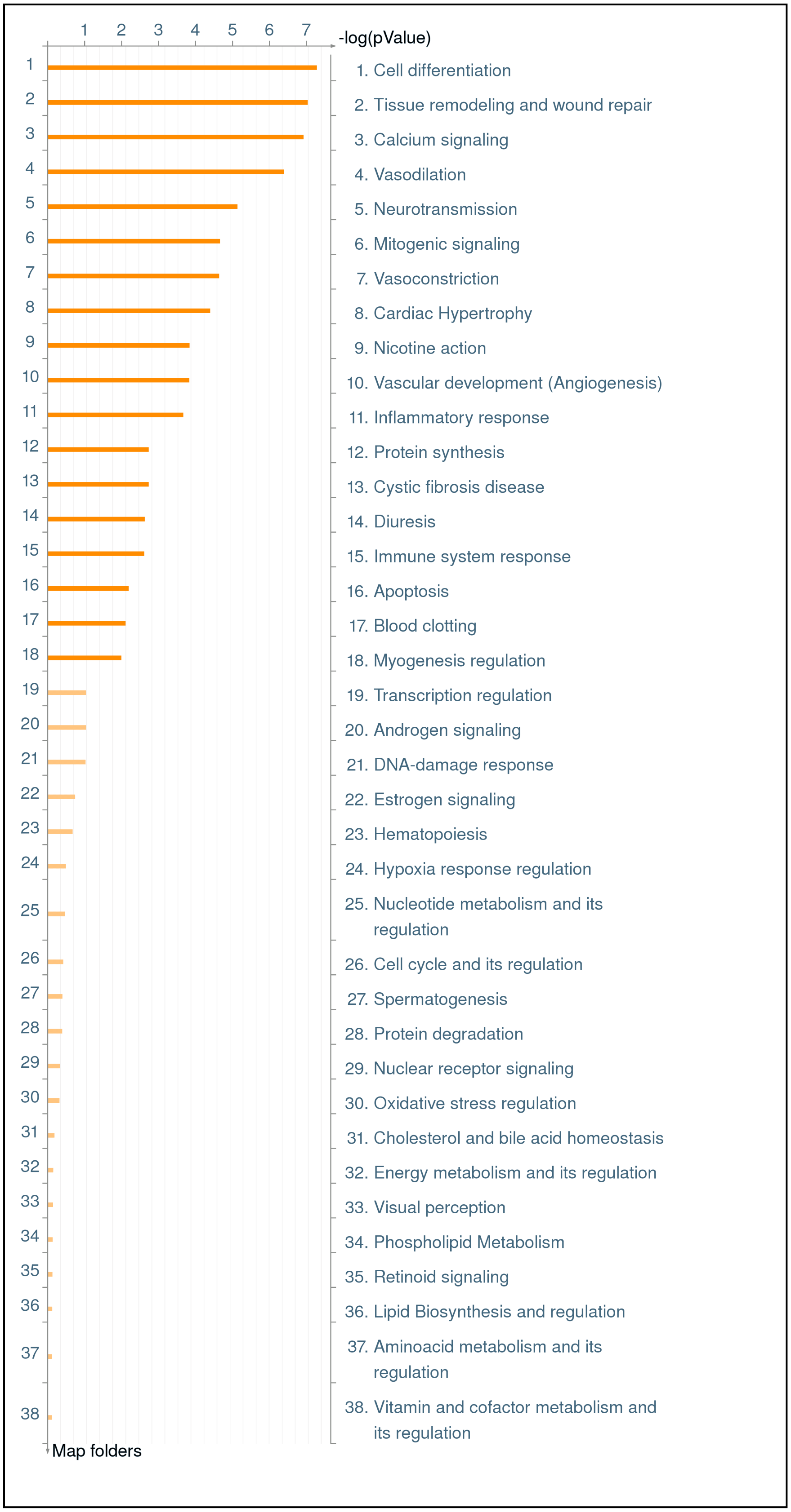

Figure S6. Significantly enriched biologically relevant pathways of genes that distinguish Ju/'hoan and Yoruba. Significant ancestry informative markers that distinguish the hunter-gatherer Ju/'hoan in our study from a representative of agriculturally-defined Yoruba, defined by a –log P value greater than 5, that are most significantly represented within genes grouped within biologically relevant pathways.
