# Supplementary material for: Complex Patterns of Genomic Admixture within Southern Africa
Source: PLoS Genet. 2013 Mar 14;9(3):e1003309. doi: 10.1371/journal.pgen.1003309 (PMC3597481; doi:10.1371/journal.pgen.1003309)
Supplement: Figure S7 — Regional distribution of the study sample and autosomal ancestral contributions. While the ‘African non-Khoesan’ (Bantu and Sandawe) contribution increases from west to east, the ‘European’ contribution decreases. Although maternal ‘Khoesan’ contributions decrease from west to east, autosomal analyses, although showing highest frequencies in the western populations, shows decreased frequency of contributions at the southern point and an inverse ‘Asian’ contribution. (PDF) [file pgen.1003309.s007.pdf]

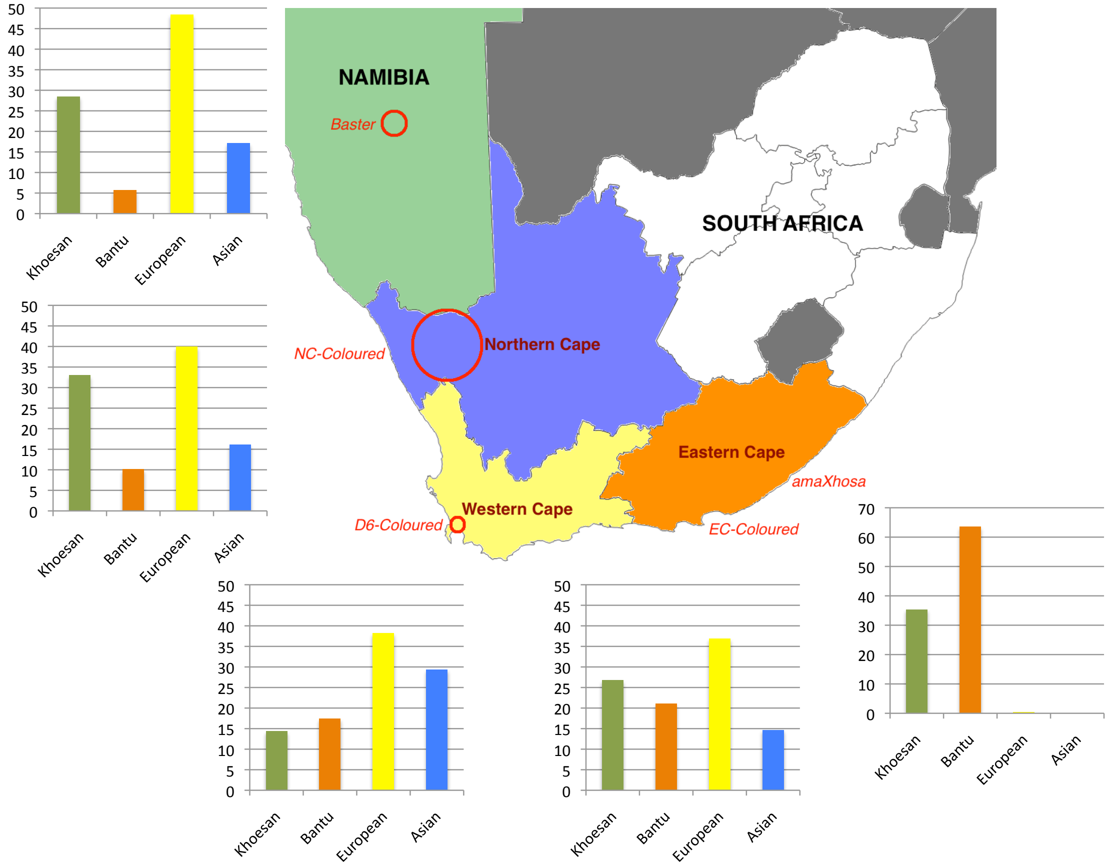

Figure S7. Regional distribution of the study sample and autosomal ancestral contributions. While the 'African non-Khoesan' (Bantu and Sandawe) contribution increases from west to east, the 'European' contribution decreases. Although maternal 'Khoesan' contributions decrease from west to east, autosomal analyses, although showing highest frequencies in the western populations, shows decreased frequency of contributions at the southern point and an inverse 'Asian' contribution.
