# Supplementary material for: Complex Patterns of Genomic Admixture within Southern Africa
Source: PLoS Genet. 2013 Mar 14;9(3):e1003309. doi: 10.1371/journal.pgen.1003309 (PMC3597481; doi:10.1371/journal.pgen.1003309)
Supplement: Figure S8 — Ju/'hoan and Yoruba ancestral contributions to the !Xun and amaXhosa. STRUCTURE analysis using the 2687 Ju/'hoan-Yoruba AIMs assuming two (estimated log likelihood of probability of data −160813.9) and three (−161042.6) population clusters (10000 burn-ins and 20000 iterations). K = 3 is also presented in the main text (Figure 4A). (PDF) [file pgen.1003309.s008.pdf]

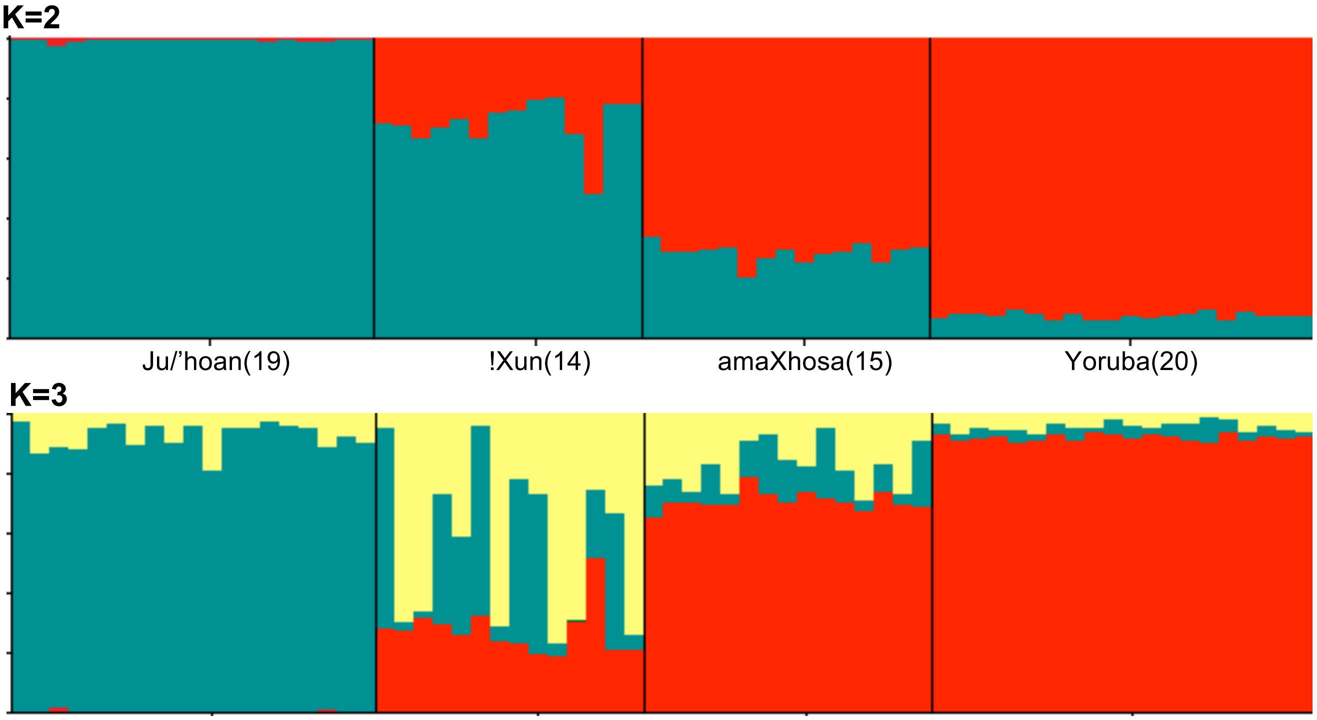

Figure S8. Ju/'hoan and Yoruba ancestral contributions to the !Xun and amaXhosa. STRUCTURE analysis using the 2687 Ju/'hoan-Yoruba AIMs assuming two (estimated log likelihood of probability of data -160813.9) and three (-161042.6) population clusters (10000 burn-ins and 20000 iterations). K=3 is also presented in the main text (**Figure 4A**).
