# Supplementary material for: Complex Patterns of Genomic Admixture within Southern Africa
Source: PLoS Genet. 2013 Mar 14;9(3):e1003309. doi: 10.1371/journal.pgen.1003309 (PMC3597481; doi:10.1371/journal.pgen.1003309)
Supplement: Figure S9 — Structure analysis for 9,317 LD pruned autosomal SNPs using K = 2 to K = 5 for ancestral populations with 5000 burn-in iterations and 10000 iterations. Indian populations listed as 1 to 10 in accordance with Figure 5B. (PDF) [file pgen.1003309.s009.pdf]

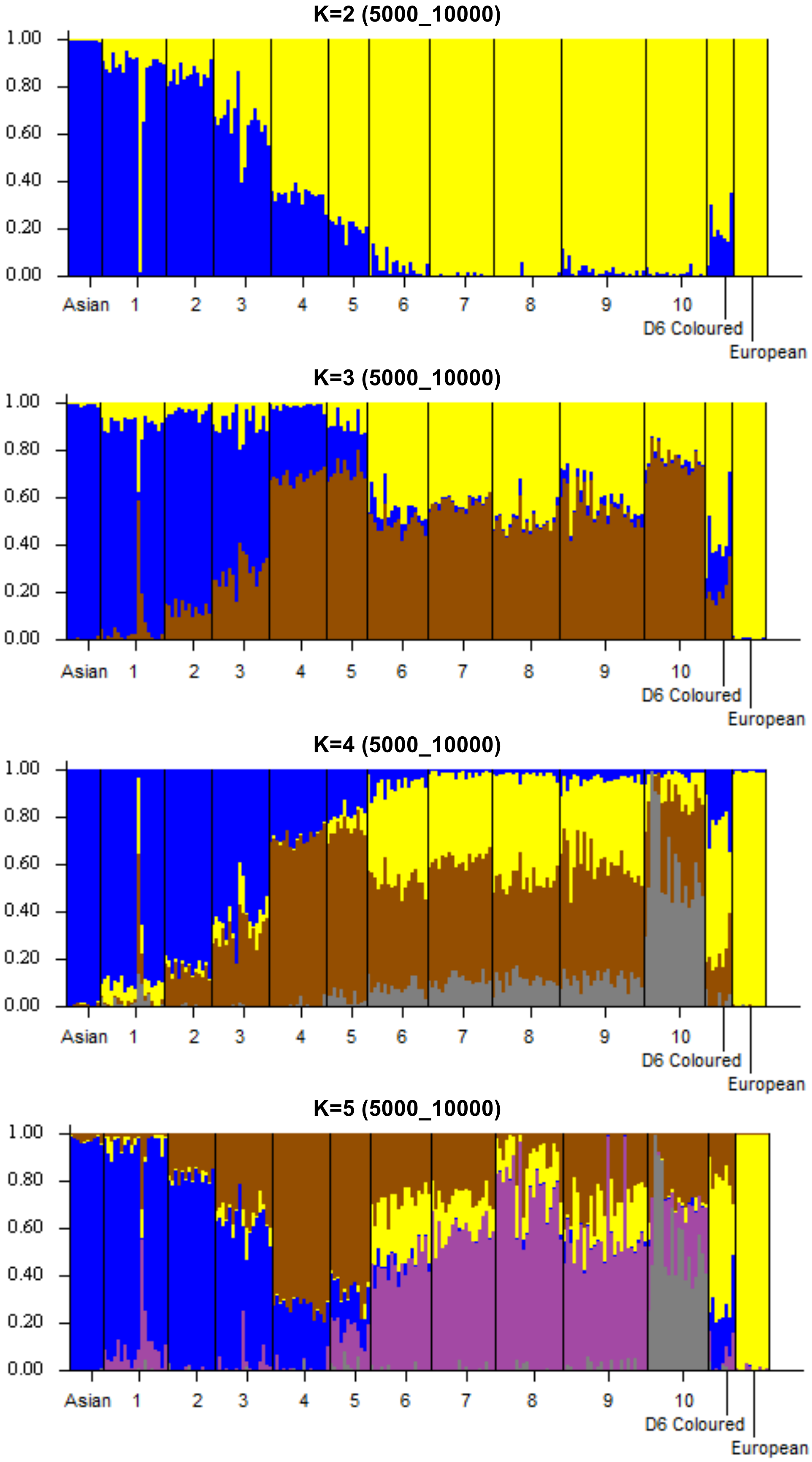

Figure S9. Structure analysis for 9,317 LD pruned autosomal SNPs using K=2 to K=5 for ancestral populations with 5000 burn-in iterations and 10000 iterations. Indian populations listed as 1 to 10 in accordance with Figure 5B.
