# Supplementary material for: Complex Patterns of Genomic Admixture within Southern Africa
Source: PLoS Genet. 2013 Mar 14;9(3):e1003309. doi: 10.1371/journal.pgen.1003309 (PMC3597481; doi:10.1371/journal.pgen.1003309)
Supplement: Figure S10 — Principal component analysis (PCA) including 9,317 LD pruned autosomal markers for (A) D6 Coloured, Han Chinese, European and 10 Indian populations (B) D6 Coloured, Han Chinese and five Indian populations (GC1 and GC2) that were not impacted by colonization (C) D6 Coloured, European and five Indian populations (GC3 and GC4) that were impacted by colonization. (PDF) [file pgen.1003309.s010.pdf]

A

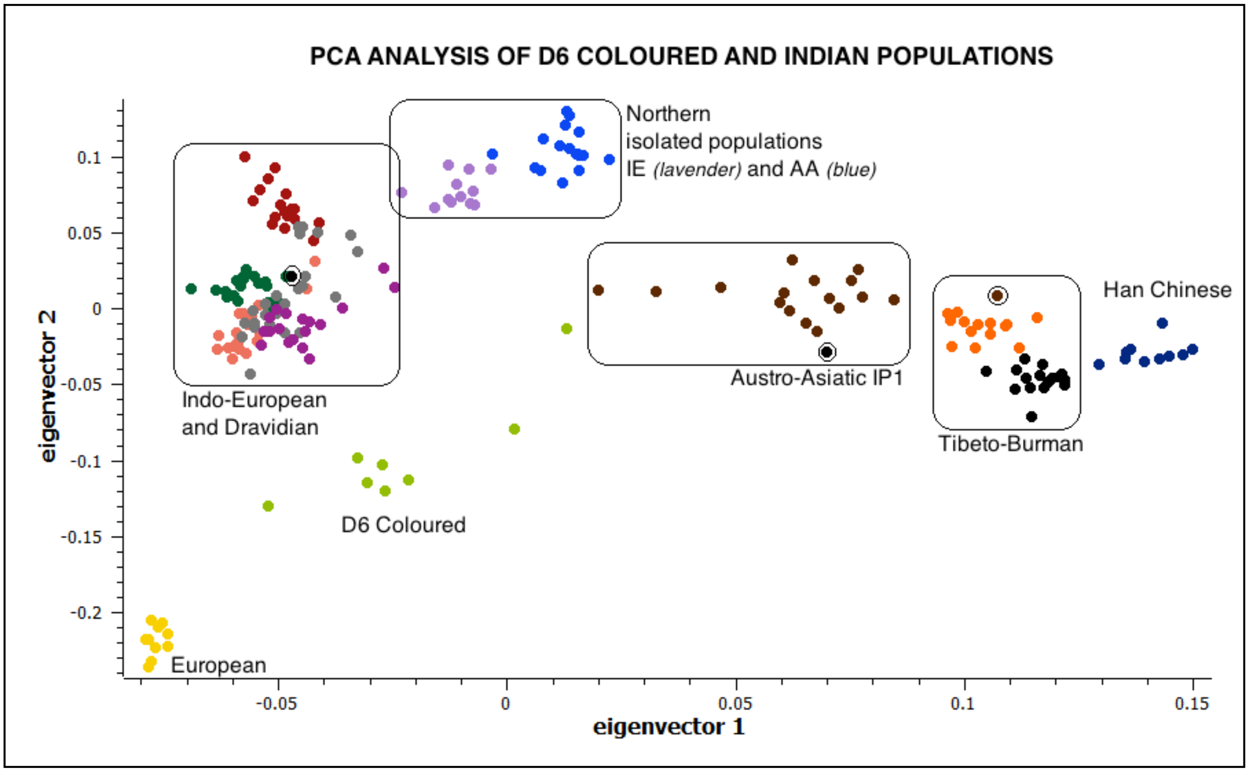
PCA ANALYSIS OF D6 COLOURED AND INDIAN POPULATIONS
Northern
isolated populations
IE (lavender) and AA (blue)
Indo-European
and Dravidian
Austro-Asiatic IP1
Han Chinese
Tibeto-Burman
D6 Coloured
European
eigenvector 2
eigenvector 1
0.1
0.05
0
-0.05
-0.1
-0.15
-0.2
-0.05
0
0.05
0.1
0.15

B

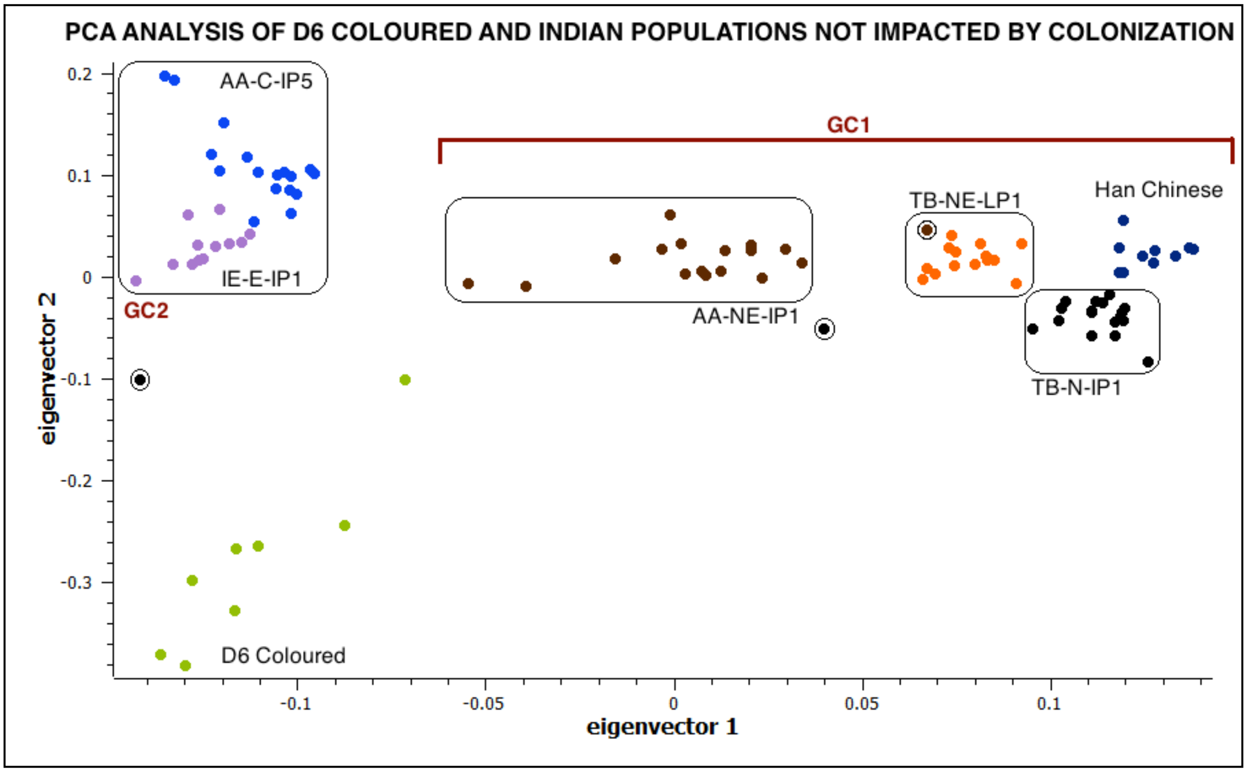
PCA ANALYSIS OF D6 COLOURED AND INDIAN POPULATIONS NOT IMPACTED BY COLONIZATION
AA-C-IP5
IE-E-IP1
GC2
GC1
AA-NE-IP1
TB-NE-LP1
Han Chinese
TB-N-IP1
D6 Coloured
eigenvector 2
eigenvector 1
0.2
0.1
0
-0.1
-0.2
-0.3
-0.1
-0.05
0
0.05
0.1

C

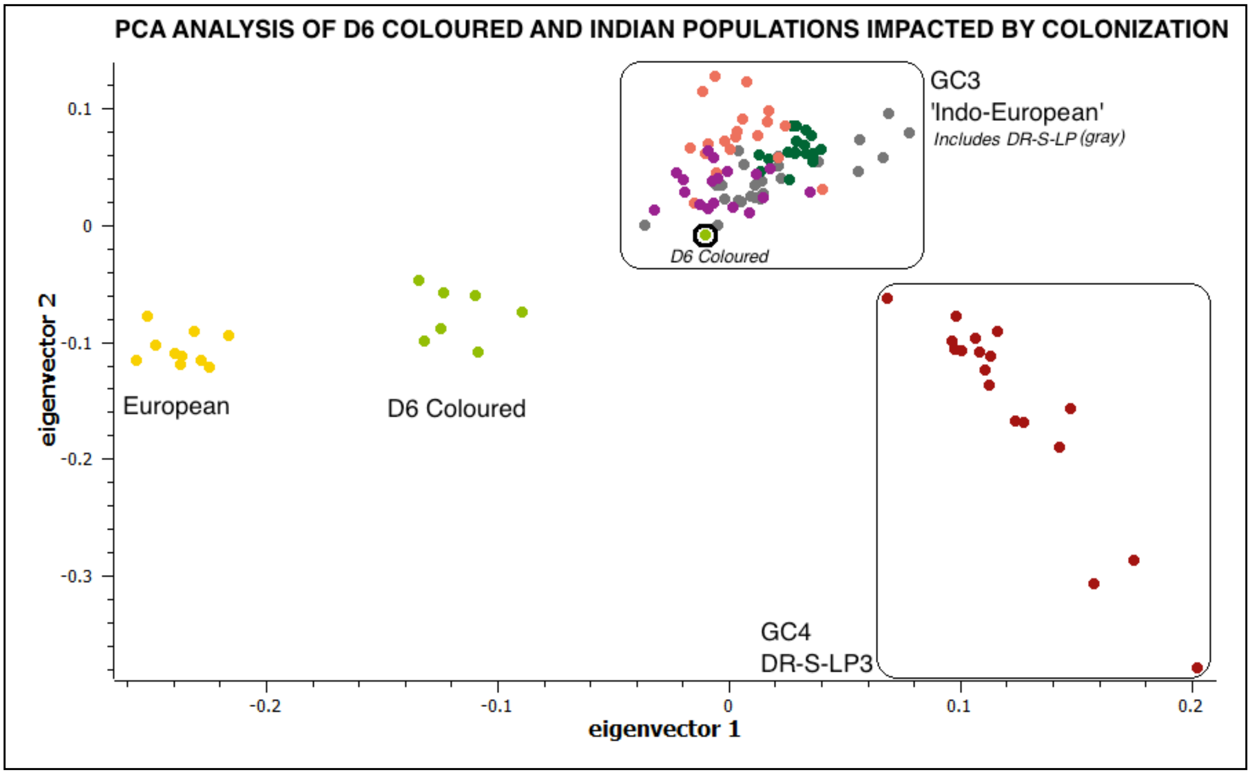

Figure S10. Principal component analysis (PCA) including 9,317 LD pruned autosomal markers for (A) D6 Coloured, Han Chinese, European and 10 Indian populations (B) D6 Coloured, Han Chinese and five Indian populations (GC1 and GC2) that were not impacted by colonization (C) D6 Coloured, European and five Indian populations (GC3 and GC4) that were impacted by colonization.
