# Supplementary material for: Complex Patterns of Genomic Admixture within Southern Africa
Source: PLoS Genet. 2013 Mar 14;9(3):e1003309. doi: 10.1371/journal.pgen.1003309 (PMC3597481; doi:10.1371/journal.pgen.1003309)
Supplement: Table S1 — Mitochondrial DNA haplogroups screened. (PDF) [file pgen.1003309.s011.pdf]

Table S1. Mitochondrial DNA haplogroups screened.

mt-DNA Haplogroup	Markers
L0	C3516A
L0d	T4232C
L0k	G4541A
L0a	G5231A and G11176A
L2	T10115C
L3’4	T3594C
L3	A1018G
M	C10400T
N	G8701A and G10398A
