# Supplementary material for: Complex Patterns of Genomic Admixture within Southern Africa
Source: PLoS Genet. 2013 Mar 14;9(3):e1003309. doi: 10.1371/journal.pgen.1003309 (PMC3597481; doi:10.1371/journal.pgen.1003309)
Supplement: Table S2 — Y-chromosomal haplogroup and subclade representation within the Baster and Coloured sample from our study. (PDF) [file pgen.1003309.s012.pdf]

Table S2. Y-chromosomal haplogroup and subclade representation within the Baster and Coloured sample from our study.

Sample	Region	Y-haplogroup	Marker	SNP
COL5	D6	E1b1a1a1g1	P277	rs16980558
COL7	D6	E1b1b1b1a2	M183	rs2032600
COL4	D6	O1a2	M50	rs2032632
COL6	D6	R1a1a1	M417	rs17316771
COL1	D6	R1b1a2	S10	rs2058276
COL3	D6	R1b1a2	S10	rs2058276
COL8	D6	R1b1a2a1a1a	M405	rs16981293
COL2	D6	R2	PS4	rs34551924
COL14	EC	E1b1a1a1f1a	M191	rs2032590
COL10	EC	E1b1a1a1g1	P277	rs16980558
COL12	EC	E1b1a1a1g1	P277	rs16980558
COL13	EC	J2b2	M241	rs8179022
COL11	EC	N1c	M46	rs34442126
COL9	EC	R1b1a2	S10	rs2058276
COL15	EC	R1b1a2	S10	rs2058276
COL21	NC	E2b	M54	rs2032620
COL18	NC	G2a3	S126	rs34134567
COL17	NC	I2a2a	P219	rs17221964
COL20	NC	R1a1a1	M417	rs17316771
COL19	NC	R1b1a2a1a1a5c1	S170	rs34283263
COL16	NC	R2	PS4	rs34551924
BAS10	REH	E1b1a1a1g1	P277	rs16980558
BAS5	REH	I2a	L460	rs7892855
BAS13	REH	I2a2a	P219	rs17221964
BAS4	REH	J	S34	rs17316547
BAS2	REH	R1a1a1	M417	rs17316771
BAS9	REH	R1a1a1	M417	rs17316771
BAS12	REH	R1b1a2	S10	rs2058276
BAS1	REH	R1b1a2a1a1a	M405	rs16981293
BAS6	REH	R1b1a2a1a1a	M405	rs16981293
BAS8	REH	R1b1a2a1a1a5c1	S170	rs34283263
BAS3	REH	R1b1a2a1a1b3	S28	rs1236440
BAS7	REH	R1b1a2a1a1b3	S28	rs1236440
BAS11	REH	R2	PS4	rs34551924

COL, Coloured; BAS, Baster
D6, District Six; EC, Eastern Cape; NC, Northern Cape and REH, Rehoboth
