# Supplementary material for: Complex Patterns of Genomic Admixture within Southern Africa
Source: PLoS Genet. 2013 Mar 14;9(3):e1003309. doi: 10.1371/journal.pgen.1003309 (PMC3597481; doi:10.1371/journal.pgen.1003309)
Supplement: Table S3 — Multiple regression GAMOVA defines the contribution of variation explained by population identifiers. (PDF) [file pgen.1003309.s013.pdf]

Table S3. Multiple regression GAMOVA defines the contribution of variation explained by population identifiers.

Population identifier	N	Cumulative PVE	P-value	Ancestral representation
European	175	0.074	< 0.001	European
Han Chinese	44	0.117	< 0.001	Asian
Yoruba	90	0.132	< 0.001	African non-Khoesan
Ju/'hoan *(this study)*	19	0.141	< 0.001	African Khoesan
Hadza	17	0.15	< 0.001	-
#Khomani	31	0.157	< 0.001	-
Baster *(this study)*	30	0.165	< 0.001	-
Mbuti	13	0.172	< 0.001	-
Biaka	22	0.179	< 0.001	-
!Xun *(this study)**	13	0.185	< 0.001	-
Coloured *(this study)*	25	0.19	< 0.001	-
Sandawe	28	0.195	< 0.001	-
Maasai	30	0.198	< 0.001	-
Luhya	36	0.201	< 0.001	-
amaXhosa *(this study)*	15	0.202	1	-

* Recently admixed individual (NF2) removed from analysis.
