# Supplementary material for: Complex Patterns of Genomic Admixture within Southern Africa
Source: PLoS Genet. 2013 Mar 14;9(3):e1003309. doi: 10.1371/journal.pgen.1003309 (PMC3597481; doi:10.1371/journal.pgen.1003309)
Supplement: Table S4 — Fixed Ju/'hoan-specific alleles. Of the 2,687 ancestry informative Ju/'hoan-Yoruba markers (AIMs), 32 were found to be fixed in the Ju/'hoan (n = 19), 16 occurring in gene regions of potential functional relevance. Of the 16 fixed Ju/'hoan alleles, 13 are defined as ancestral against the Pan (Chimp) genome. (PDF) [file pgen.1003309.s014.pdf]

Table S4. **Fixed Ju/'hoan-specific alleles.** Of the 2,687 ancestry informative Ju/'hoan-Yoruba markers (AIMs), 32 were found to be fixed in the Ju/'hoan (n=19), 16 occurring in gene regions of potential functional relevance. Of the 16 fixed Ju/'hoan alleles, 13 are defined as ancestral against the *Pan* (Chimp) genome.

**Gene relevant SNP**	**Chr**	**Position[1]**	**Gene**	**Location**	**Function[2]**	**Genetic Association Class (Phenotype)[3]**	***Pan* allele**	**Southern African Populations (our data)**			**HapMap Populations (published)**		
								**Ju/'hoan (n=19) 'Fixed'**	!Xun (n=14)	Xhosa (n=15)	**Yoruba (n=90)**	Asian (n=44)	European (n=175)
rs3767216	1	20012927 [20140340]	*RNF186*	Downstream	Ring finger protein involved in protein ubiquitination and diverse cellular processes.	*Immune (Ulcerative Colitis)*	**A**	**A**	A(0.714) G(0.286)	A(0.500) G(0.500)	A(0.400) G(0.600)	A(0.364) G(0.636)	A(0.686) G(0.314)
rs6662137	1	216608682 [218542059]	*TGFB2*	Intronic	Cellular functions including regulating proliferation, differentiation, adhesion, and migration in many cell types. Suppresses the effects of interleukin dependent T-cell tumors.	*Cancer (Ovarian / Breast); Infection (Hepatitis C / Malaria); Immune (Asthma / MS / Lupus / Celiac disease); Cardiovascular (atherosclerosis); Chemical dependency (alcohol); Metabolic (obesity / bone density); Aging (kidney again); Vision (myopia); Developmental (cleft palate)*	**G**	**G**	G(0.857) T(0.143)	G(0.700) T(0.300)	G(0.589) T(0.411)	G(0.352) T(0.648)	G(0.306) T(0.694)
rs13432082	2	230609891 [230901647]	*SLC16A14*	3'UTR	Proton-linked monocarboxylate transporter.	None	**A**	**A**	A(0.964) G(0.036)	A(0.767) G(0.233)	A(0.344) G(0.656)	A(0.852) G(0.148)	A(0.731) G(0.269)
rs5011271	6	46250398 [46142439]	*ENPP5*	Promoter	Ectonucleotide pyrophosphatase /phosphodiesterase 5 with hydrolase activity. May play a role in neuronal cell communication.	None	**A**	**A**	A(0.893) G(0.107)	A(0.833) G(0.167)	A(0.561) G(0.439)	A(0.636) G(0.364)	A(0.620) G(0.380)
rs10253875	7	1059515 [1092989]	*C7orf50*	Intronic	Protein binding and unknown function.	None	**A**	**A**	**A 'Fixed'**	A(0.667) G(0.333)	A(0.439) G(0.561)	**A 'Fixed'**	**A 'Fixed'**
rs10263183	7	83614853 [83776917]	*SEMA3A*	Intronic	Chemorepulsive agent, inhibiting axonal outgrowth, or a chemoattractive agent, stimulating the growth of apical dendrites. Vital for normal neuronal pattern development.	*Chemical dependency (tobacco use); Neurological (Alzheimer's)*	**T**	**C 'Derived'**	**C 'Fixed'**	C(0.733) T(0.267)	C(0.617) T(0.383)	C(0.784) T(0.216)	C(0.863) T(0.137)
rs1464798	7	138982540 [139332000]	*HIPK2*	Intronic	Protein kinase. Co-repressor of several transcription factors. Inhibits cell growth and promotes apoptosis. Involved in transcriptional activation of TP53 and TP73.	*Chemical dependency (tobacco use)*	**T**	**T**	T(0.893) C(0.107)	T(0.733) C(0.267)	T(0.556) C(0.444)	T(0.102) C(0.898)	T(0.429) C(0.571)
rs1043421	11	76514209 [76836561]	*CAPN5*	3'UTR	Calpain, a calcium-dependent cysteine proteases involved in signal transduction in a variety of cellular processes.	*Reproduction (Polycystic Ovarian Syndrome); Cardiovascular (atherosclerosis / blood pressure); Neurological (Cognitive performance); Metabolic (obesity)*	**T**	**T**	**T 'Fixed'**	T(0.833) A(0.167)	T(0.589) A(0.411)	T(0.739) A(0.261)	T(0.774) A(0.226)
rs10841661	12	20876099 [20984832]	*SLCO1B3*	Intronic	Solute carrier organic anion transporter. Normally expressed in the liver and uptakes large, non-polar drugs and hormones from the portal vein.	*Cancer (Leukemia / lymphoma / prostate / neoplasms); Infection (Tuberculosis); Metabolic (Hyperbilirubinemia); Renal (Kidney failure); Developmental (height); Pharmacogenomic (mycophenolic acid pharmacokines / drug-related genes /* docetaxel elimination / paclitaxel pharmacokinetics)	**T**	**T**	T(0.857) C(0.143)	T(0.700) C(0.300)	T(0.589) C(0.411)	T(0.341) C(0.659)	T(0.440) C(0.560)

rs9566462	13	39067423 [40169423]	*LHFP*	Intronic	Tetraspan transmembrane protein. A fusion partner of *HMGIC* gene in a translocation-associated lipoma t(12;13).	None	**C**	**T** **'Derived'**	T(0.857) C(0.143)	T(0.633) C(0.367)	T(0.589) C(0.411)	T(0.205) C(0.795)	T(0.177) C(0.823)
rs2070342	14	23615206 [24545366]	*CPNE6*	Coding (synonymous)	A brain-specific member of the copine family, which is composed of calcium-dependent membrane-binding proteins. Contains two N-terminal C2 domains, and one von Willebrand factor A domain. May have a role in synaptic plasticity.	None	**G**	**G**	G(0.964) A(0.036)	G(0.733) A(0.267)	G(0.550) A(0.450)	G(0.386) A(0.614)	G(0.386) A(0.614)
rs17126976	14	90255620 [91185867]	*TTC7B*	Intronic	Tetratricopeptide repeat protein 7B. Participates in host cellular shutoff mediated by the Chikungunya virus (CHIKV) viral nonstructural protein nsP2 (previously reported for other Old World alphaviruses).	None	**A**	**A**	A (0.846) G (0.154)	A(0.767) G(0.233)	A(0.589) G(0.411)	A(0.955) G(0.045)	A(0.799) G(0.201)
rs16966391	15	31264514 [33477222]	*FMN1*	Intronic	Formin protein involved in the formation of adherens junction and the polymerization of linear actin cables.	*Cancer (prostate cancer): Chemical dependency (tobacco use); Developmental (cleft palate); Alcohol associated pancreatitis*	**T**	**C** **'Derived'**	C(0.643) T(0.357)	C(0.800) T(0.200)	C(0.533) T(0.467)	C(0.852) T(0.148)	C(0.940) T(0.060)
rs1425287	15	58783129 [60995837]	*RORA*	Intronic	Orphan nuclear receptor that binds DNA as a monomer to hormone response elements (HREs) containing a single core motif half-site followed by a short AT-rich sequence. Regulates numerous genes involved in lipid metabolism and in cerebellum and photoreceptor development.	*Psycological (depression / bipolar disporder); Chemical dependency (tobacco use); Sleep disorders; Pharmacogenomic (response to citalopram treatment)*	**G**	**G**	G(0.929) A(0.071)	G(0.867) A(0.133)	G(0.633) A(0.367)	G(0.773) A(0.227)	G(0.709) A(0.291)
rs7239719	18	4194614 [4204614]	*DLGAP1*	Intronic	Disks large-associated protein is found at high concentrations in synaptic junctions and forms part of the postsynaptic scaffold in neuronal cells.	*Chemical dependency (tobacco use)*	**T**	**T**	T(0.893) C(0.107)	T(0.833) C(0.167)	T(0.628) C(0.372)	T(0.943) C(0.057)	T(0.905) C(0.095)
rs57531768	22	41964694 [43634750]	*SCUBE1*	Intronic	Cell surface glycoprotein in platelets and endothelial cells and may have an important function in vascular biology.	None	**T**	**T**	T(0.929) C(0.071)	T(0.733) C(0.267)	T(0.567) C(0.433)	T(0.943) C(0.057)	T(0.983) C(0.017)

[1] Position as defined by the HapMap Project (http://hapmap.ncbi.nlm.nih.gov/) and the Illumina array content (http://www.illumina.com/science/icontroldb.ilmn). The position in brackets as defined by Genome Build 37.3 used by NCBI dbSNP (http://www.ncbi.nlm.nih.gov/projects/SNP/) and the UCSC Genome Browser (http://genome.ucsc.edu/).
[2]Information for protein function was obtained using NCBI Gene (http://www.ncbi.nlm.nih.gov/gene), NCBI OMIM (http://www.ncbi.nlm.nih.gov/omim) and GeneCardsV3 (http://www.genecards.org/).
[3] Previous gene-based association as defined by the Genetic Association Database (http://geneticassociationdb.nih.gov/), note the association is only loci relevant and not directly associated with the specific SNP listed.
