# Supplementary material for: Complex Patterns of Genomic Admixture within Southern Africa
Source: PLoS Genet. 2013 Mar 14;9(3):e1003309. doi: 10.1371/journal.pgen.1003309 (PMC3597481; doi:10.1371/journal.pgen.1003309)
Supplement: Table S5 — Alleles with a highly significant −log10P>14 Ju/'hoan versus Yoruba population-specific association. (PDF) [file pgen.1003309.s015.pdf]

Table S5. Alleles with a highly significant –log10P>14 Ju/'hoan versus 'Yoruba population-specific association.

-log10 P	SNP	Chr	Position[1]	Gene	Location	Genetic Association Class (Phenotype)[2]	Pan allele	Allele frequencies					
								Southern African Populations (our study)			HapMap populations (Illumina iControl Database)		
								Ju/'hoan (n=19)	!Xun (n=14)	Xhosa (n=15)	Yoruba (n=90)	Asian (n=44)	European (n=175)
							Ethnic / Geographical classification	*Khoesan*		*African*		*Non-African*	
							Subsistence based classification	*Forager*		*Agriculturalist*			
20.03726772	rs2594762	4	159102581 [158883131]	–	–	-	**T**	T(0.750) C(0.250)	T(0.143) C(0.857)	T(0.100) C(0.900)	T(0.006) C(0.994)	T(0.023) C(0.977)	T(0.109) C(0.891)
19.12504517	rs2477414	9	33544121 [33554121]	*ANKRD18B*	Intronic	None	**T**	T(0.658) C(0.342)	T(0.464) C(0.536)	T(0.233) C(0.767)	**C(1)**	T(0.045) C(0.955)	T(0.129) C(0.871)
18.01377702	rs12695746	3	143130049 [141647359]	*ATP1B3*	Downstream	None	**G**	G(0.500) T(0.500)	G(0.714) T(0.286)	G(0.900) T(0.100)	**G(1)**	G(0.795) T(0.205)	G(0.854) T(0.146)
17.81036587	rs283722	8	128379719 [128310537]	*LOC727677*	Intronic	None	**G**	G(0.368) A(0.632)	G(0.786) A(0.214)	G(0.867) A(0.133)	G(0.989) A(0.011)	G(0.977) A(0.023)	**G(1)**
17.73368261	rs12410304	1	243885443 [245818820]	*KIF26B*	Intronic	*Chemical dependency (tobacco use)*	**G**	G(0.421) A(0.579)	G(0.536) A(0.464)	G(0.867) A(0.133)	**G(1)**	G(0.602) A(0.398)	G(0.943) A(0.057)
17.62553771	rs13379392	14	87408696 [88338943]	*GALC*	Intronic	*Neurological (Krabbe disease)*	**G**	G(0.526) T(0.474)	G(0.357) T(0.643)	G(0.033) T(0.967)	G(0.011) T(0.989)	G(0.057) T(0.943)	G(0.425) T(0.575)
17.39638358	rs2723269	12	38916054 [40629787]	*LRRK2*	Intronic	*Chemical dependency (tobacco use); Neurological (Parkinson's disease); Immune (Crohn disease); Infection (Leprosy)*	**A**	A(0.368) T(0.632)	A(0.786) T(0.214)	A(0.967) T(0.033)	A(0.994) T(0.006)	A(0.636) T(0.364)	A(0.937) T(0.063)
17.25799595	rs13032068	2	111539508 [111823037]	*ACOXL*	Intronic	*Cancer (Chronic lymphocytic leukemia)*	**A**	A(0.632) G(0.368)	A(0.214) G(0.786)	A(0.133) G(0.867)	A(0.017) G(0.983)	A(0.091) G(0.909)	A(0.095) G(0.905)
17.23160021	rs10502172	11	112704356 [113199146]	*TTC12*	Intronic	*Chemical dependency (nicotine / alcohol dependency)*	**C**	C(0.611) T(0.389)	C(0.800) T(0.200)	C(0.750) T(0.250)	**C(1)**	C(0.943) T(0.057)	C(0.549) T(0.451)
15.94274955	rs17753447	1	234748204 [236681581]	*LGALS8*	5'UTR	None	**A**	A(0.711) G(0.289)	A(0.321) G(0.679)	A(0.200) G(0.800)	A(0.044) G(0.956)	A(0.045) G(0.955)	A(0.169) G(0.831)
15.81839562	rs13216265	6	15925857 [15817878]	–	–	-	**T**	T(0.806) C(0.194)	T(0.500) C(0.500)	T(0.267) C(0.733)	T(0.067) C(0.933)	T(0.091) C(0.909)	T(0.163) C(0.837)
15.80162901	rs12432055	14	35559090 [36489339]	–	–	-	**C**	C(0.579) T(0.421)	C(0.929) T(0.071)	C(0.967) T(0.033)	**C(1)**	C(0.898) T(0.102)	C(0.954) T(0.046)
15.72915232	rs2552241	8	5841292 [5853884]	–	–	-	**C**	C(0.237) T(0.763)	C(0.286) T(0.714)	C(0.767) T(0.233)	C(0.917) T(0.083)	C(0.761) T(0.239)	C(0.489) T(0.511)
15.59791193	rs6998580	8	94964119 [94894943]	–	–	-	**T**	T(0.605) C(0.395)	T(0.321) C(0.679)	T(0.067) C(0.933)	T(0.022) C(0.978)	T(0.080) C(0.920)	T(0.423) C(0.577)
15.18605643	rs348609	5	40296686 [40260929]	*U1*[3]	Upstream	-	**C**	C(0.316)T(0.684)	C(0.714) T(0.286)	C(0.700) T(0.300)	C(0.950) T(0.050)	C(0.966) T(0.034)	C(0.797) T(0.203)

15.14420001	rs6839885	4	101917906 [101698883]	–	–	-	**T**	T(0.393)C(0.607)	T(0.077) C(0.923)	**C(1)**	**C(1)**	T(0.102) C(0.898)	T(0.257) C(0.743)
15.10634209	rs10279013	7	72789825 [73151889]	*ABHD11*	Intronic	None	**C**	C(0.500) T(0.500)	C(0.750) T(0.250)	C(0.967) T(0.033)	C(0.989) T(0.011)	**C(1)**	**C(1)**
15.07562289	rs17005571	3	69390632 [69307942]	*FRMD4B*	Intronic	*Chemical dependency (tobacco use); Cardiovascular (Heart failure); Immune (Celiac disease)*	**T**	T(0.632) C(0.368)	T(0.643) C(0.357)	T(0.933) C(0.067)	**T(1)**	T(0.875) C(0.125)	T(0.983) C(0.017)
14.74817121	rs4854190	2	3086042 [3107035]	*AK095310*	Intronic	None	**G**	G(0.684) A(0.316)	G(0.893) A(0.107)	**G(1)**	**G(1)**	G(0.807) A(0.193)	G(0.920) A(0.080)
14.74817121	rs2132276	7	97664276 [97826340]	*LMTK2*	Intronic	*Cancer (Prostate cancer)*	**G**	G(0.684) A(0.316)	G(0.786) A(0.214)	**G(1)**	**G(1)**	G(0.909) A(0.091)	G(0.454) A(0.546)
14.60808907	rs2836716	21	39157929 [40236059]	–	–	-	**A**	A(0.553) G(0.447)	A(0.429) G(0.571)	**G(1)**	A(0.028) G(0.972)	**G(1)**	A(0.080) G(0.920)
14.54275662	rs13092634	3	192334655 [190851961]	–	–	-	**A**	A(0.395)G (0.605)	A(0.607) G(0.393)	A(0.967) G(0.033)	A(0.956) G(0.044)	**A(1)**	A(0.951) G(0.049)
14.36201865	rs10789536	11	106072227 [106567017]	*GUCY1A2*	Intronic	*Chemical dependency (tobacco use)*	**A**	A(0.263)G (0.737)	A(0.536) G(0.464)	A(0.800) G(0.200)	A(0.911) G(0.089)	A(0.341) G(0.659)	A(0.363) G(0.637)
14.24534597	rs12666164	7	37932726 [37966201]	*EPDR1*	Intronic	*Chemical dependency (tobacco use); Cancer (Prostate cancer)*	**T**	T(0.605) C(0.395)	T(0.857) C(0.143)	T(0.900) C(0.100)	T(0.989) C(0.011)	T(0.852) C(0.148)	T(0.680) C(0.320)
14.17566315	rs7867486	9	99350355 [100310534]	*TMOD1*	Intronic	None	**G**	G(0.553) A(0.447)	G(0.714) A(0.286)	G(0.933) A(0.067)	**G(1)**	G(0.784) A(0.216)	G(0.783) A(0.217)
14.07888701	rs2497401	13	84749880 [85851879]	–	–	-	**T**	T(0.737) C(0.263)	T(0.321) C(0.679)	T(0.167) C(0.833)	T(0.083) C(0.917)	T(0.045) C(0.955)	T(0.100) C(0.900)

[1] Position as defined by the HapMap Project (http://hapmap.ncbi.nlm.nih.gov/) and the Illumina array content (http://www.illumina.com/science/icontroldb.ilmn). The position in brackets as defined by Genome Build 37.3 used by NCBI dbSNP (http://www.ncbi.nlm.nih.gov/projects/SNP/) and the UCSC Genome Browser (http://genome.ucsc.edu/).

[2] Previous gene-based association as defined by the Genetic Association Database (http://geneticassociationdb.nih.gov/)

[3] The U1 gene is a uridine-rich small nuclearribo-nucleoprotein (snRNP), which carries out many essential functions via interactions of their RNA and protein components with the pre-mRNA, specifically, they mediate the recognition and subsequent pairing of the 5′ and 3′ splice sites of an intron [58].
