# Supplementary material for: Complex Patterns of Genomic Admixture within Southern Africa
Source: PLoS Genet. 2013 Mar 14;9(3):e1003309. doi: 10.1371/journal.pgen.1003309 (PMC3597481; doi:10.1371/journal.pgen.1003309)
Supplement: Table S6 — Of the 2687 Ju/'hoan versus Yoruba AIMs (−log10P>5) 33 are nonsynonymous variants and are ordered in descending order from most significant. (PDF) [file pgen.1003309.s016.pdf]

Table S6. Of the 2687 Ju/'hoan versus Yoruba AIMs (–log10P>5) 33 are nonsynonymous variants and are ordered in descending order from most significant.

-log10 P	SNP (rs#)	Chr	Position[1]	Gene	Gene product (GO)[2]	Base change	AA change	SIFT prediction (score)[3]	Genetic Association Class (Phenotype)[4]	Pan[5]	Ju/'hoan (n=19)	Yoruba[6] (n=90)
12.07595565	rs2272662	8	145610534 [145639726]	*SLC39A4*	Zinc transporter (BP: cellular zinc iron homeostasis / transmembrane transport)	A>G	T332A	Tolerated (0.15)	*Reproduction (spontaneous abortion)*	A	A(0.5363) G(0.4737)	A(0.9889) G(0.0111)
11.29070609	rs28434777	7	150122089 [150491156]	*TMEM176B*	Transmembrane protein (BP: cell differentiation / organ morphogenesis)	T>C	T70A	Tolerated (0.14)	none	T	T(0.5) C(0.5)	T(0.95) C(0.05)
10.81051294	rs479661	2	169429623 [169721377]	*NOSTRIN*	Nitric oxide (NO) synthase trafficker (BP: negative regulation of transcription / endocytosis / NO metabolic process)	T>C	G473E	Tolerated (0.77)	*Chemical dependency (tobacco use)*<br>*Psychology (autism)*	T	T(0.7368) C(0.2632)	T(0.9944) C(0.0056)
10.31647493	rs3911730	18	66022323 [67871343]	*RTTN*	Rotatin (BP: determination of left/right symmetry)	A>C	S126A	Tolerated (0.85)	none	A	A(0.4474) C(0.5526)	A(0.9333) C(0.0667)
8.941965582	rs3809045	11	118549542 [119044332]	*NLRX1*	Nod-like receptor (BP: innate immune response)	G>T	R125L	Tolerated (0.07)	none	G	G(0.7632) T(0.2368)	G(1)
8.807859407	rs857725	1	156874559 [158607935]	*SPTA1*	Erythrocyte spectrin alpha chain (BP: actin filament organization)	T>G	K1693Q	Tolerated (0.59)	*Hematological (erythrocyte phenotype)*<br>*Chemical dependency (tobacco use)*<br>*Cardiovascular (hypertension)*	T	T(0.4737) G(0.5263)	T(0.9222) G(0.0778)
8.506041418	rs36074897	19	17332346 [17471346]	*PLVAP*	Plasmalemma vesicle-associated protein (MF: protein homodimerization activity)	G>A	A438V	Tolerated (0.41)	none	G	G(0.8158) A(0.1842)	G(1)
8.506041418	rs5030980	16	66074446 [67516945]	*AGRP*	Agouti-related protein (BP: feeding behavior / hormone-mediated signaling pathway)	G>A	A67T	Tolerated (0.95)	*Metabolic (body mass)*<br>*Psychology (anorexia nervosa)*	G	G(0.8158) A(0.1842)	G(1)
8.502829686	rs28689661	16	79793826 [81236325]	*PKD1L2*	Polycystic kidney disease protein (BP: neuropeptide signaling pathway)	A>C	V308G	Damaging (0)	*Chemical dependency (smoking cessation)*<br>*Psychological (conduct disorder or ADHD)*<br>*Metabolic (high-density lipoprotein cholesterol)*	A	A(0.5789) C(0.4211)	A(0.9444) C(0.0556)
7.971233109	rs11592585	10	133805417 [133955427]	*JAKMIP3*	Janus kinase and microtubule-interacting protein (MF: kinase binding / microtubule binding)	A>G	M493V	Tolerated (0.31)	none	A	A(0.7632) G(0.2368)	A(0.9944) G(0.0056)
7.941568766	rs5036	17	39694471 [42338945]	*SLC4A1*	Anion transport protein (BP: ion transport or homeostasis)	A>G	K56E	Tolerated (1.0)	*Cardiovascular (blood pressure)*<br>*Renal*<br>*Hemotological (malarial anemia, spherocytosis, priapism)*<br>*Metabolic (Hyperparathyroidism)*	ND	A(0.5263) G(0.4737)	A(0.9222) G(0.0778)
7.535865391	rs11575216	12	55041724 [56755457]	*APOF*	Apolipoprotein (BP: lipid metabolic process / lipid transport)	C>G	A178G	Tolerated (0.39)	*Infection (Hepatitis C remission)*<br>*Neurological (Alzheimer's)*<br>*Cardiovascular (coronary artery disease)*	G	C(0.7632) G(0.2368)	C(0.9833) G(0.0167)
7.407783096	rs34156839	19	11346552 [11485552]	*SWSAP1*	ATPase SWIM-type zinc finger associated protein (BP: ATP catabolic process / double-strand break repair via homologous recombination / protein stabilization)	C>T	L45F	Tolerated (0.18)	none	C	C(0.6316) T(0.3684)	C(0.9556) T(0.0444)
7.319832012	rs12440118	15	40531386 [42744094]	*ZFP106*	Zinc finger protein (BP: insulin receptor signaling pathway)	A>G	W103R	Tolerated (0.92)	*Chemical dependency (Tobacco use)*	ND	A(0.8421) G(0.1579)	A(1)
7.258842632	rs1893963	18	26903040 [28649042]	*DSC2*	Desmocollin (BP: homophilic cell adhesion)	A>G	I776V	Tolerated (1.0)	*Cardiovascular (arythmia, cardiomyopathy)*	G	A(0.0789) G(0.9211)	A(0.5944) G(0.4056)

6.805988329	rs9932495	16	23629366 [23721865]	*ERN2*	Endoplasmic reticulum to nucleus-signaling (BP: mRNA processing)	C>T	V117I	Tolerated (0.46)	*Chemical dependency (Tobacco use)*	C	C(0.6316) T(0.3684)	C(0.9389) T(0.0611)
6.460162224	rs17690300	2	75746619 [75893111]	*GCFC2* *(MRPL19)**	GC-rich sequence DNA-binding factor (BP: regulation of transcription)	T>G	E724D (intronic)	Not scored	none *[Chemical dependency (tobacco use)]*	T	T(0.8611) G(0.1389)	T(1)
6.447033053	rs12863734	13	85268572 [86370571]	*SLITRK6*	SLIT and NTRK-like protein or Leucine-rich repeat-containing protein (BP: axonogenesis)	G>A	L25F	Tolerated (0.09)	none	A	G(0.7632) A(0.2368)	G(0.9833) A(0.0167)
6.411250347	rs35928055	7	23704416 [23737891]	*C7orf46*	Uncharacterized protein	A>G	S240G	Tolerated (0.35)	none	A	A(0.7895) G(0.2105)	A(0.9833) G(0.0167)
6.210532664	rs690514	17	70374188 [72862593]	*FDXR*	Mitochondrial adrenodoxin oxidoreductase like (BP: NADPH oxidation)	G>A	R123Q	Tolerated (0.31)	none	G	G(0.7632) A(0.2368)	G(0.9889) A(0.0111)
6.155237791	rs12305038	12	20413519 [20522252]	*PDE3A*	cGMP-inhibited 3',5'-cyclic phosphodiesterase (multiple processes)	G>A	D12N	Tolerated (0.23)	*Reproduction (male infertility)* *Developmental (height)* *Cardiovascular (QT interval, aortic root size, cardiovascular disease)*	G	G(0.8421) A(0.1579)	G(0.3889) A(0.6111)
6.150397387	rs2231524	8	141594509 [141525327]	*CHRAC1*	Chromatin assembly complex protein (BP: chromatin remodeling / DNA replication / histone acetylation)	A>G	H126R	Tolerated (0.75)	none	A	A(0.8684) G(0.1316)	A(1)
6.150397387	rs12671170	7	20160504 [20193979]	*MACC1*	Metastasis-associated in colon cancer protein (BP: regulation of transcription / positive regulation of cell division)	T>G	E728A	Tolerated (0.06)	none	G	T(0.8684) G(0.1316)	T(1)
6.150397387	rs34255002	1	21104047 [21231460]	*EIF4G3*	Eukaryotic translation initiation factor (BP: RNA metabolic process / cytokine-mediated signaling pathway / regulation of translation / spermatogenesis)	T>G	Q500H	Damaging (0.03)	*Chemical dependency (Tobacco use)*	T	T(0.8684) G(0.1316)	T(1)
5.664800375	rs35371972	17	36830744 [39577218]	*KRT37*	Keratin protein (MF: Structural molecule activity)	G>A	S421F	Damaging (0)	none	G	G(0.7222) A(0.2778)	G(0.9611) A(0.0389)
5.544786783	rs2748421	8	142586404 [142517222]	*FLJ43860*	Uncharacterized protein	T>C	Y10H	Tolerated (1.0)	none	C	T(0.4737) C(0.5263)	T(0.8611) C(0.1389)
5.469163162	rs3744550	17	10147320 [10206595]	*MYH13*	Myosin (BP: cellular response to starvation / muscle contraction)	T>C	H1862R	Tolerated (1.0)	*Chemical dependency (Tobacco use)* *Neurological (Alzheimer's)*	C	T(0.5263) C(0.4737)	T(0.8722) C(0.1278)
5.331572094	rs2306513	17	30484629 [33460516]	*NLE1*	Notchless Drosophila homolog (BP: Notch signaling / inner cell mass cell differentiation)	A>G	Y114H	Tolerated (0.08)	none	A	A(0.8333) G(0.1667)	A(0.9889) G(0.0111)
5.325684488	rs17513722	2	61920937 [62067433]	*FAM161A*	Protein FAM (BP: response to stimulus / visual perception)	T>C	I236V	Tolerated (0.5)	none	T	T(0.8158) C(0.1842)	T(0.9833) C(0.0167)
5.226628042	rs2291157	4	5771922 [5721021]	*EVC*	Ellis-van Creveld syndrome protein (BP: Skeletal system development)	T>G	Q74P	Tolerated (0.12)	*Chemical dependency (Tobacco use)* *Psychology (suicide)* *Developmental (cleft palate)*	T	T(0.6842) G(0.3158)	T(0.9556) G(0.0444)
5.208574822	rs17121403	1	100108565 [100335977]	*AGL*	Glycogen debranching enzyme (BP: glycogen biosynthesis)	A>G	Q229R	Tolerated (0.66)	*Chemical dependency (Tobacco use)* *Metabolic (glycogen storage disease)*	A	A(0.4737) G(0.5263)	A(0.8427) G(0.1573)
5.058425123	rs16902381	5	35694996 [35659239]	*SPEF2*	Sperm flagellar (BP: nucleobase-containing metabolic process)	G>A	R366K	Tolerated (0.07)	*Chemical dependency (Tobacco use)*	G	G(0.7895) A(0.2105)	G(0.9722) A(0.0278)
5.042427081	rs2289080	2	233114422 [233406178]	*CHRNG*	Acetylcholine receptor subunit gamma (BP: muscle contraction / signal transduction / synaptic transmission / transport)	G>A	A149T	Tolerated (0.19)	*Chemical dependency (Tobacco use)* *Cardiovascular (cardiovascular disease)* *Psychological (multiple disorders)*	G	G(0.8421) A(0.1579)	G(0.9889) A(0.0111)

[1]Chromosome location as per Illumina array Reference Assembly Build 36.3 or in brackets Assembly GRCh37.p5 Build 37.3; [2]GO, gene ontology (http://amigo.geneontology.org/): BP=Biological process or MF=molecular function; [3]SIFT prediction (http://sift.jcvi.org); [4]Positive or suggested gene-phenotype classification as per the Genetic Association Database (http://geneticassociationdb.nih.gov/), no associations were SNP specific; [5] Chimpanzee (*Pan troglodytes*) allele, derived from the genome sequence (version PanTro2; http://genome.ucsc.edu); [6]Data derived from the Illumina iControl Database (www.illumina.com); * Overlap between genes although intronic in *MRPL19*. This gene was included as it has been previously associated with tobacco use. ND (not determined)
