# Supplementary material for: Complex Patterns of Genomic Admixture within Southern Africa
Source: PLoS Genet. 2013 Mar 14;9(3):e1003309. doi: 10.1371/journal.pgen.1003309 (PMC3597481; doi:10.1371/journal.pgen.1003309)
Supplement: Table S7 — Ju/'hoan versus Yoruba differentiating AIMs located within genes ranked according to significance of genes enriched for biological processes. (PDF) [file pgen.1003309.s017.pdf]

Table S7. Ju/'hoan versus Yoruba differentiating AIMs located within genes ranked according to significance of genes enriched for biological processes.

#	Biological Processes	pValue	Ratio	
1	nervous system development	1.021E-31	233	2226
2	synaptic transmission	2.141E-26	111	756
3	transmission of nerve impulse	2.933E-25	117	851
4	multicellular organismal signaling	2.933E-25	117	851
5	generation of neurons	6.267E-25	160	1414
6	neurogenesis	7.589E-25	166	1499
7	multicellular organismal development	8.846E-25	372	4791
8	system development	1.285E-24	331	4089
9	cell development	1.842E-24	169	1553
10	neuron development	7.110E-24	120	920
11	neuron differentiation	8.468E-24	133	1087
12	anatomical structure development	1.047E-23	353	4519
13	cell differentiation	3.586E-23	264	3047
14	developmental process	4.101E-23	391	5228
15	cell-cell signaling	5.658E-23	136	1149
16	cellular developmental process	8.439E-23	268	3130
17	multicellular organismal process	1.398E-22	486	7028
18	cell projection organization	1.943E-22	120	956
19	regulation of system process	2.310E-20	92	661
20	behavior	1.099E-19	86	605
21	regulation of synaptic transmission	1.936E-19	58	307
22	regulation of transmission of nerve impulse	5.159E-19	60	333
23	central nervous system development	1.128E-18	110	929
24	anatomical structure morphogenesis	1.593E-18	199	2230
25	regulation of localization	1.640E-18	163	1680
26	ion transport	4.175E-18	120	1080
27	ion transmembrane transport	4.648E-18	79	558
28	neuron projection development	9.646E-18	95	761
29	regulation of neurological system process	1.664E-17	60	357
30	signaling	2.400E-17	386	5525
31	regulation of signaling	4.302E-17	196	2251
32	calcium ion transport	5.509E-17	46	226
33	regulation of membrane potential	6.930E-17	52	285
34	neuron projection morphogenesis	6.941E-17	86	670
35	cell projection morphogenesis	7.827E-17	92	747
36	cell communication	1.179E-16	393	5707
37	cell part morphogenesis	2.769E-16	92	762
38	locomotory behavior	3.236E-16	42	199
39	regulation of developmental process	3.345E-16	156	1671
40	regulation of multicellular organismal process	4.204E-16	187	2157
41	regulation of biological quality	4.729E-16	230	2860
42	regulation of ion transport	4.913E-16	62	405
43	cell morphogenesis involved in neuron differentiation	5.794E-16	83	656
44	divalent metal ion transport	1.995E-15	48	267
45	regulation of nucleotide metabolic process	2.127E-15	78	608
46	divalent inorganic cation transport	3.634E-15	48	271
47	regulation of transport	4.193E-15	129	1314
48	axon guidance	6.997E-15	65	463
49	axonogenesis	8.879E-15	78	624
50	cognition	8.998E-15	47	267

Green number, number of genes that contain Ju/'hoan or Yoruba AIMs
Red number, total number of genes in the biological process gene ontology identifier
