# Supplementary material for: Complex Patterns of Genomic Admixture within Southern Africa
Source: PLoS Genet. 2013 Mar 14;9(3):e1003309. doi: 10.1371/journal.pgen.1003309 (PMC3597481; doi:10.1371/journal.pgen.1003309)
Supplement: Table S8 — Ju/'hoan versus Yoruba differentiating AIMs located within genes ranked according to significance of genes enriched for molecular functions. (PDF) [file pgen.1003309.s018.pdf]

Table S8. Ju/'hoan versus Yoruba differentiating AIMs located within genes ranked according to significance of genes enriched for molecular functions.

#	Molecular Functions	pValue	Ratio	
1	binding	1.972E-14	693	13874
2	ion transmembrane transporter activity	2.116E-14	83	796
3	protein binding	6.622E-13	481	8906
4	acetylcholine receptor binding	8.071E-13	11	15
5	substrate-specific transmembrane transporter activity	2.182E-12	87	930
6	calcium ion binding	3.266E-12	75	750
7	transmembrane transporter activity	1.019E-11	91	1022
8	ion binding	2.134E-11	271	4462
9	metal ion binding	2.143E-11	268	4400
10	cation binding	2.586E-11	270	4449
11	ion channel activity	3.079E-11	50	418
12	gated channel activity	4.866E-11	43	330
13	substrate-specific channel activity	6.649E-11	50	427
14	substrate-specific transporter activity	9.385E-11	92	1082
15	channel activity	1.514E-10	51	451
16	passive transmembrane transporter activity	1.639E-10	51	452
17	cation channel activity	7.444E-10	38	293
18	PTB domain binding	1.405E-09	8	11
19	cation transmembrane transporter activity	5.179E-09	58	606
20	transporter activity	7.150E-09	100	1320
21	nucleoside-triphosphatase regulator activity	8.672E-09	49	477
22	GTPase regulator activity	9.193E-09	48	463
23	enzyme activator activity	1.385E-08	48	469
24	channel regulator activity	1.485E-08	18	86
25	voltage-gated channel activity	5.692E-08	28	208
26	voltage-gated ion channel activity	5.692E-08	28	208
27	guanyl-nucleotide exchange factor activity	2.173E-07	25	183
28	calcium channel regulator activity	2.308E-07	10	30
29	small GTPase regulator activity	3.416E-07	33	295
30	actin binding	7.953E-07	37	365
31	adenyl ribonucleotide binding	1.429E-06	108	1627
32	adenyl nucleotide binding	2.064E-06	108	1640
33	peptidase activator activity	2.987E-06	11	47
34	ATP binding	3.293E-06	105	1599
35	active transmembrane transporter activity	4.305E-06	37	392
36	calmodulin binding	5.141E-06	21	163
37	enzyme regulator activity	6.651E-06	78	1112
38	voltage-gated cation channel activity	1.118E-05	20	158
39	cytoskeletal protein binding	1.795E-05	50	636
40	polysaccharide binding	2.173E-05	24	221
41	pattern binding	2.173E-05	24	221
42	calcium channel activity	2.272E-05	14	90
43	voltage-gated calcium channel activity	2.440E-05	8	30
44	purine ribonucleotide binding	2.827E-05	121	2001
45	ribonucleotide binding	2.891E-05	121	2002
46	Rho guanyl-nucleotide exchange factor activity	3.148E-05	13	81
47	Ras guanyl-nucleotide exchange factor activity	3.710E-05	15	106
48	purine nucleotide binding	4.017E-05	121	2017
49	glycosaminoglycan binding	4.512E-05	22	202
50	transmembrane receptor protein tyrosine phosphatase activity	4.529E-05	6	17

Green number, number of genes that contain Ju/'hoan or Yoruba AIMs
Red number, total number of genes in the molecular functions gene ontology identifier
