# Supplementary material for: Complex Patterns of Genomic Admixture within Southern Africa
Source: PLoS Genet. 2013 Mar 14;9(3):e1003309. doi: 10.1371/journal.pgen.1003309 (PMC3597481; doi:10.1371/journal.pgen.1003309)
Supplement: Table S9 — Ju/'hoan versus Yoruba differentiating AIMs located within genes ranked according to significance of genes enriched for cellular localization. (PDF) [file pgen.1003309.s019.pdf]

Table S9. Ju/'hoan versus Yoruba differentiating AIMs located within genes ranked according to significance of genes enriched for cellular localization.

#	Cellular Localizations	pValue	Ratio	
1	cell periphery	2.646E-19	356	4856
2	plasma membrane	3.437E-18	347	4767
3	synapse	4.483E-16	74	542
4	plasma membrane part	1.220E-14	194	2346
5	synapse part	1.666E-12	56	409
6	intrinsic to plasma membrane	3.228E-12	130	1453
7	membrane	5.901E-12	539	9022
8	cell projection	2.881E-11	119	1329
9	integral to plasma membrane	3.687E-11	123	1396
10	neuron projection	1.141E-09	75	744
11	cell junction	1.323E-09	73	718
12	dendrite	2.251E-09	50	414
13	synaptic membrane	4.635E-09	33	217
14	ion channel complex	1.164E-08	33	225
15	proteinaceous extracellular matrix	2.407E-08	47	405
16	postsynaptic density	7.925E-08	23	132
17	dendritic spine head	7.925E-08	23	132
18	presynaptic membrane	2.203E-07	15	63
19	basement membrane	2.609E-07	19	100
20	neuron spine	3.031E-07	27	186
21	dendritic spine	3.031E-07	27	186
22	neuronal cell body	3.208E-07	43	387
23	cell projection part	3.224E-07	65	700
24	extracellular matrix	3.884E-07	49	472
25	cell body	5.389E-07	44	408
26	cell leading edge	8.410E-07	33	269
27	membrane part	1.872E-06	418	7239
28	membrane fraction	2.408E-06	95	1221
29	cation channel complex	2.485E-06	22	148
30	insoluble fraction	2.617E-06	98	1273
31	extracellular matrix part	7.809E-06	26	207
32	postsynaptic membrane	9.073E-06	24	184
33	intrinsic to membrane	9.192E-06	366	6306
34	calcium channel complex	1.393E-05	9	32
35	dendritic shaft	3.596E-05	10	44
36	membrane raft	4.013E-05	25	214
37	axon	5.620E-05	33	328
38	basolateral plasma membrane	6.019E-05	32	315
39	integral to membrane	8.547E-05	351	6160
40	apical part of cell	9.689E-05	32	323
41	voltage-gated calcium channel complex	9.954E-05	7	24
42	cytoskeleton	1.030E-04	123	1836
43	synaptosome	1.343E-04	19	152
44	apical plasma membrane	1.733E-04	26	248
45	adherens junction	2.358E-04	22	198
46	anchoring junction	3.427E-04	23	217
47	ruffle	1.191E-03	15	127
48	cell fraction	1.203E-03	108	1680
49	cell cortex	1.562E-03	20	199
50	Golgi apparatus	2.095E-03	77	1149

Green number, number of genes that contain Ju/'hoan or Yoruba AIMs
Red number, total number of genes in the cellular localization gene ontology identifier
