# Supplementary material for: Complex Patterns of Genomic Admixture within Southern Africa
Source: PLoS Genet. 2013 Mar 14;9(3):e1003309. doi: 10.1371/journal.pgen.1003309 (PMC3597481; doi:10.1371/journal.pgen.1003309)
Supplement: Table S10 — Ju/'hoan versus Yoruba differentiating AIMs located within genes ranked according to significance of genes enriched for disease associations. (PDF) [file pgen.1003309.s020.pdf]

Petersen DC, et al. **Complex Patterns of Genomic Admixture within Southern Africa**

Table S10. Ju/'hoan versus Yoruba differentiating AIMs located within genes ranked according to significance of genes enriched for disease associations.

#	Diseases	pValue	Ratio	
1	Multiple Sclerosis	2.079E-39	286	2205
2	Demyelinating Diseases	2.354E-39	292	2279
3	Demyelinating Autoimmune Diseases, CNS	2.973E-39	286	2209
4	Autoimmune Diseases of the Nervous System	3.195E-38	287	2248
5	Autoimmune Diseases	1.151E-30	337	3121
6	Nervous System Diseases	8.846E-26	440	4799
7	Immune System Diseases	8.443E-22	432	4867
8	Depressive Disorder	1.648E-18	127	918
9	Mood Disorders	1.816E-18	146	1131
10	Psychiatry and Psychology	2.681E-18	279	2820
11	Depressive Disorder, Major	2.826E-18	126	913
12	Mental Disorders	3.905E-18	277	2800
13	Tobacco Use Disorder	6.226E-18	65	319
14	Respiratory Hypersensitivity	4.198E-14	134	1125
15	Asthma	1.417E-13	132	1119
16	Bronchial Diseases	4.978E-13	135	1174
17	Lung Diseases, Obstructive	1.520E-12	137	1216
18	Bipolar Disorder	1.081E-11	90	693
19	Affective Disorders, Psychotic	1.268E-11	90	695
20	Hypersensitivity	1.491E-11	151	1430
21	Hypersensitivity, Immediate	1.853E-11	137	1258
22	Amyloid Neuropathies, Familial	6.477E-10	9	11
23	Lewy Body Disease	1.820E-09	18	54
24	Amyloid Neuropathies	2.447E-09	9	12
25	Schizophrenia	3.001E-09	107	976
26	Schizophrenia and Disorders with Psychotic Features	3.769E-09	107	980
27	Cerebral Amyloid Angiopathy	1.197E-08	14	36
28	Epilepsy	2.658E-08	51	361
29	Mental Disorders Diagnosed in Childhood	4.242E-08	55	409
30	Pancytopenia	5.643E-08	6	6
31	Attention Deficit Disorder with Hyperactivity	1.047E-07	18	68
32	Substance-Related Disorders	1.165E-07	86	783
33	Alzheimer disease, early onset	2.160E-07	12	32
34	Memory Disorders	6.101E-07	11	29
35	Anxiety Disorders	9.144E-07	30	183
36	Trisomy	1.113E-06	17	71
37	Chromosome Duplication	1.113E-06	17	71
38	Autistic Disorder	1.127E-06	25	138
39	Attention Deficit and Disruptive Behavior Disorders	1.183E-06	18	79
40	Child Development Disorders, Pervasive	1.295E-06	25	139
41	Cerebral Arterial Diseases	1.307E-06	14	50
42	Gliosis	1.331E-06	11	31
43	Monosomy	1.417E-06	6	8
44	Seizures	2.429E-06	26	153
45	Behavior and Behavior Mechanisms	3.591E-06	50	411
46	Neurologic Manifestations	4.337E-06	118	1273
47	Glucose Metabolism Disorders	5.080E-06	162	1883
48	Cocaine-Related Disorders	1.083E-05	10	31
49	Muscular Diseases	1.253E-05	55	489
50	Aortic Aneurysm, Thoracic	1.379E-05	8	20

Green number, number of genes that contain Ju/'hoan or Yoruba AIMs
Red number, total number of genes in the diseases gene ontology identifier
