# Supplementary material for: Complex Patterns of Genomic Admixture within Southern Africa
Source: PLoS Genet. 2013 Mar 14;9(3):e1003309. doi: 10.1371/journal.pgen.1003309 (PMC3597481; doi:10.1371/journal.pgen.1003309)
Supplement: Table S11 — Allele sharing distances within (gray blocks) and between populations using identity-by-state (IBS) and including Fst distance values. (PDF) [file pgen.1003309.s021.pdf]

Table S11. Allele sharing distances within (gray blocks) and between populations using identity-by-state (IBS) and including Fst distance values.

		**Ju/'hoan**	**!Xun**	#Khomani	Mbuti	Biaka	**Xhosa**	Hadza	Sandawe	**Baster**	**Coloured**	Luhya	Yoruba	Maasai	European	Han Chinese
		Fst-values														
Ju/'hoan	**IBS distance**	**0.224**	0.047	0.045	0.116	0.096	0.091	0.133	0.098	0.093	0.092	0.101	0.104	0.105	0.151	0.188
!Xun		0.24	**0.245**	0.042	0.102	0.078	0.071	0.114	0.077	0.078	0.075	0.077	0.079	0.082	0.13	0.169
#Khomani		0.249	0.256	**0.254**	0.273	0.275	0.275	0.283	0.283	0.28	0.282	0.309	0.285	0.287	0.304	0.311
Mbuti		0.266	0.269	0.09	**0.237**	0.268	0.091	0.127	0.091	0.104	0.099	0.085	0.09	0.094	0.145	0.182
Biaka		0.27	0.271	0.066	0.08	**0.255**	0.057	0.1	0.062	0.076	0.07	0.051	0.051	0.062	0.115	0.151
Xhosa		0.274	0.272	0.056	0.278	0.274	**0.268**	0.284	0.281	0.062	0.054	0.033	0.032	0.282	0.1	0.138
Hadza		0.281	0.281	0.099	0.284	0.283	0.089	**0.247**	0.075	0.093	0.087	0.076	0.082	0.075	0.123	0.162
Sandawe		0.283	0.281	0.061	0.284	0.283	0.047	0.284	**0.273**	0.049	0.043	0.033	0.038	0.03	0.076	0.119
Baster		0.283	0.285	0.05	0.294	0.292	0.29	0.296	0.29	**0.277**	0.02	0.312	0.296	0.29	0.283	2.297
Coloured		0.285	0.286	0.05	0.293	0.291	0.288	0.295	0.289	0.281	**0.283**	0.313	0.293	0.29	0.286	0.296
Luhya		0.285	0.282	0.062	0.282	0.278	0.274	0.284	0.28	0.056	0.047	**0.272**	0.019	0.28	0.085	0.124
Yoruba		0.287	0.283	0.066	0.284	0.278	0.274	0.287	0.283	0.061	0.051	0.296	**0.268**	0.283	0.091	0.129
Maasai		0.289	0.287	0.065	0.288	0.285	0.045	0.286	0.281	0.045	0.039	0.028	0.034	**0.277**	0.068	0.112
European		0.311	0.309	0.104	0.313	0.31	0.307	0.308	0.301	0.034	0.037	0.322	0.309	0.299	**0.267**	0.299
Han Chinese		0.317	0.315	0.144	0.319	0.316	0.314	0.315	0.31	0.091	0.083	0.332	0.316	0.309	0.098	**0.246**
