# Supplementary material for: Complex Patterns of Genomic Admixture within Southern Africa
Source: PLoS Genet. 2013 Mar 14;9(3):e1003309. doi: 10.1371/journal.pgen.1003309 (PMC3597481; doi:10.1371/journal.pgen.1003309)
Supplement: Table S12 — Contribution of four ancestral populations to the amaXhosa, Baster and Coloured populations displayed as a percentage for both the average and range distributions. (PDF) [file pgen.1003309.s022.pdf]

Table S12. Contribution of four ancestral populations to the amaXhosa, Baster and Coloured populations displayed as a percentage for both the average and range distributions.

	*N**	African Khoesan	African Non-Khoesan	European	Asian
**!Xun**	13				
Average		77.1%	20.5%	0.9%	-
Range		72% - 82.3%	16.8% - 27%	0% - 3.4%	
**!Xun NF2**	1				
Recent Bantu admixture		52.2%	47%	0.1%	-
**#Khomani**	31				
Average		70.6%	13.9%	11%	3.7%
Range		27.9% - 97.4%	2.2% - 55.8%	0% - 41.3%	0% - 19.1%
**amaXhosa**	15				
Average		35.4%	63.6%	0.5%	-
Range		26.4% - 40.3%	58.5% - 69.3%	0% -3.3%	
**Basters**	30				
Average		28.5%	5.7%	48.4%	17.1%
Range		16.9% - 41.5%	0.2% - 13.1%	37.4% - 60.6%	11.6 - 26.8%
**NC Coloured**	10				
Average		33%	10.1%	40%	16.1%
Range		20.6% - 55.8%	3.5% - 19.7%	15.7% - 57%	7.4% - 26.8%
**EC Coloured**	7				
Average		26.8%	21.1%	36.9%	14.7%
Range		16.8% - 44.8%	6.3% - 47.8%	6.7% - 53.1%	0% - 23%
**D6 Coloured**	8				
Average		14.4%	17.4%	38.2%	29.4%
Range		3.8% - 24.8%	9.2% - 29.8%	23.6% - 53.2%	16.6% - 51.6%
**Total Coloured**	25				
Average		25.3%	15.5%	38.5%	19.9%
Range		3.8% - 55.8%	3.5% - 47.8%	6.7% - 57%	0% - 51.6%

**N* = Number of samples per population group
