# Supplementary material for: Complex Patterns of Genomic Admixture within Southern Africa
Source: PLoS Genet. 2013 Mar 14;9(3):e1003309. doi: 10.1371/journal.pgen.1003309 (PMC3597481; doi:10.1371/journal.pgen.1003309)
Supplement: Table S13 — Significance of ancestral contributions within southern Africans from our study. (PDF) [file pgen.1003309.s023.pdf]

Table S13. Significance of ancestral contributions within southern Africans from our study.[1]

Population	Indian (IND)	Indonesian (IDO)	European (CEU)	Khoesan (SAN)	Yoruba (YRI)	Han Chinese (CBH)
**Total Coloured**	0.28	0.24	0.96	0.88	0.8	0.04
**D6-Coloured**	0.63	0.5	1	0.63	0.88	0
**EC-Coloured**	0.14	0.14	0.86	1	1	0
**NC-Coloured**	0.1	0.1	1	1	0.6	0.1
**Baster**	0.07	0.03	1	1	0.5	0.07
**amaXhosa**	0	0.07	0.13	1	1	0
**!Xun**	0	0.07	0	1	0.93	0
**#Khomani[2]**	0.29	0.06	0.52	1	0.52	0.03

[1] A value of 1.0 indicates that all subjects within the population identifier carried an ancestral contribution
[2] Published data, Henn et al., 2011
