# Supplementary material for: Complex Patterns of Genomic Admixture within Southern Africa
Source: PLoS Genet. 2013 Mar 14;9(3):e1003309. doi: 10.1371/journal.pgen.1003309 (PMC3597481; doi:10.1371/journal.pgen.1003309)
Supplement: Table S14 — Primer sequences and amplification conditions for gender-specific markers. (PDF) [file pgen.1003309.s024.pdf]

Table S14. Primer sequences and amplification conditions for gender-specific markers

DNA	Haplogroup	Marker	Primers
mt	L0	C3516A	3322-F 5’CTCCTACTCCTCATTGTACC-3’<br>4162-R 5'-TGAGTTGGTCGTAGCGGAAT-3'
mt	L0d	T4232C	4040-F 5'-CAACATATGACGCACTCTCC-3'<br>5995-R 5’-GTGCCTAGGACTCCAGCTCA-3’
mt	L0k	G4541A	4040-F 5'-CAACATATGACGCACTCTCC-3'<br>5995-R 5’-GTGCCTAGGACTCCAGCTCA-3’
mt	L0a	G5231A<br><br>G11176A	4838-F 5'-TCTGACATCCGGCCTGCTTC-3'<br>5995-R 5’-GTGCCTAGGACTCCAGCTCA-3’<br>10961-F 5'-CTAACTACCTGACTCCTACC-3'<br>Amp2-R 5’ TTACTTTTATTTGGAGTTGCACCA-3’
mt	L2	T10115C	9378-F 5'-TGGCGCGATGTAACACGAGA-3'<br>10380-R 5'-GTAGTCACTCATAGGCCAGA-3'
mt	L3’4	T3594C	3322-F 5’CTCCTACTCCTCATTGTACC-3’<br>4162-R 5'-TGAGTTGGTCGTAGCGGAAT-3'
mt	L3	A1018G	751-F 5'-ACAAGCATCAAGCACGCAGC-3'<br>1721-R 5'-GGCTAAGGTTGTCTGGTAGT-3'
mt	M	C10400T	10131-F 5'-CCACAACTCAACGGCTACAT-3'<br>Amp2-R 5’ TTACTTTTATTTGGAGTTGCACCA-3’
mt	N	G10398A	10131-F 5'-CCACAACTCAACGGCTACAT-3'<br>Amp2-R 5’ TTACTTTTATTTGGAGTTGCACCA-3’
Y-chr	E1b1b	M215	M215-F 5’-CATCCCATGAAATATACACAGAAAC-3’<br>M215-R 5’-AGTGAGCAGAGATTGCACCA-3’<br>M215-seq 5’-AGATTAAACTTTGATTATATTAAT-3’

Amplification conditions:
mtDNA: Roche Fast-Start PCR protocol, annealing temperature set at 60°C
Y-Chr: Roche Fast-Start PCR protocol, annealing temperature set at 56°C. The 430 bp amplicon was first excised from a 2% agarose gel (QIAquick Gel Extraction Kit, Qiagen) prior to sequencing.
